# Comparative Mitogenomics of *Pedetontus* and *Pedetontinus* (Insecta: Archaeognatha) Unveils Phylogeny, Divergence History, and Adaptive Evolution

**DOI:** 10.3390/insects16121194

**Published:** 2025-11-24

**Authors:** Wei Cen, Ting Yang, Jia-Wen Li, Dan-Na Yu, Kenneth B. Storey, Jia-Yong Zhang

**Affiliations:** 1 College of Life Sciences, Zhejiang Normal University, Jinhua 321004, China; 2 Department of Biology, Carleton University, Ottawa, ON K1S5B6, Canada; 3 Key Laboratory of Wildlife Biotechnology, Conservation and Utilization of Zhejiang Province, Zhejiang Normal University, Jinhua 321004, China

**Keywords:** mitogenomes, *Pedetontus*, *Pedetontinus*, phylogeny, divergence time, selective stress analysis

## Abstract

Archaeognatha is phylogenetically positioned as the basal lineage relative to all extant insect orders and comprises approximately 600 described species. The internal phylogenetic relationships and divergence times within this ancient order have long been a subject of scientific debate. In this study, we assembled 14 mitochondrial genomes from species within the genera *Pedetontus* and *Pedetontinus* to clarify the phylogenetic relationship and estimate divergence times within Archaeognatha. Phylogenetic analyses revealed that both Machilidae and Machilinae are paraphyletic; *Pedetontinus* included in this analysis formed a well-supported monophyletic clade, whereas the sampled *Pedetontus* species were not recovered as a monophyletic clade. Divergence time estimates indicate that Archaeognatha originated during the Late Carboniferous (301.15 Mya, 95% HPD: 298.88–303.67 Mya), with subsequent diversification spanning from the Mesozoic era to the present. The adaptive radiation of epiphytic bryophytes and potential coevolutionary interactions between plants and insects are proposed to have significantly contributed to the diversification of Archaeognatha. Based on multiple lines of evidence, we propose that the current morphological criteria for species delineation within *Pedetontus* (*Pd.*) require revision to better reflect its evolutionary history. In the branch-site model analysis, when *Pd. silvestrii*—collected from temperate regions—was designated as the foreground branch, two positively selected sites were detected at the 66th position of the *Cytb* and the 34th position of *ATP6*. When *Pd. hainanensis* and *Pd. bawanglingensis*—collected from tropical regions—were used as the foreground branches, six positively selected sites were identified at the 622nd position of *Cytb*, the 499th position of *ATP6*, and the 623rd, 873rd, 1106th, and 1141st positions of *COI*.

## Simple Summary

The evolutionary history of the order Archaeognatha has long been a subject of considerable scientific interest and debate. In this study, we utilized 14 mitochondrial genomes from *Pedetontus* and *Pedetontinus* species to investigate the phylogeny and divergence times within Archaeognatha. Integrated analyses of genetic distances, phylogenetic reconstructions, and divergence time estimations suggest that the current morphological classification system for *Pedetontus* may require revision. Furthermore, evidence of positive selection was detected in the mitochondrial protein-coding genes of these species under both temperate and tropical environmental conditions.

## 1. Introduction

Archaeognatha occupies a pivotal position at the base of the insect phylogenetic tree and comprises approximately 600 extant species, representing the sister group to all other extant insect lineages [1,2]. The order is currently recognized as comprising two extant families: Machilidae and Meinertellidae [3]. Additionally, several paleoforms, such as *Ditrigoniophthalmus*, *Charimachilis*, and *Mesomachilis*, have been proposed as early-diverging lineages that predate the split between Machilidae and Meinertellidae [4]. Although the precise phylogenetic placement of these paleoforms remains unresolved, multiple studies suggest they may retain key plesiomorphic characteristics, providing important insights into early insect evolutionary [5,6,7,8]. To date, 41 species of Archaeognatha have been documented in China [9,10,11,12,13,14,15,16,17,18,19,20,21,22,23,24,25,26,27,28,29,30], with 40 species belonging to the family Machilidae. These Machilidae species are distributed across the following genera: *Pedetontinus* (10 species), *Pedetontus* (17 species), *Haslundichilis* (2 species), *Coreamachilis* (2 species), *Metamachilis* (1 species), *Silvestrichilis* (3 species), *Allopsontus* (4 species), and *Songmachilis* (1 species). The remaining species belongs to the family Meinertellidae and is classified within the genus *Machilontus*.

Within this taxonomic framework, the family Machilidae is divided into three distinct subfamilies—Machilinae, Petrobiellinae, and Petrobiinae [31]. The genera *Pedetontus* and *Pedetontinus* are classified within the subfamily Petrobiinae. These genera share the following morphological characteristics: an unscaled antennal flagellum, large contiguous compound eyes, paired shoe-shaped ocelli situated subinferior to the compound eyes, mandibles with four apical teeth, scaled maxillary palps and thoracic legs, meso- and metacoxae each bearing a stylus, and an elongate multi-articulate ovipositor. Specifically, *Pedetontus* is characterized by fore femora that are consistently unswollen, abdominal coxites II–V/VI bearing two pairs of retractile vesicles, and a primary-type ovipositor lacking a terminal claw. In contrast, *Pedetontinus* typically exhibits unmodified fore femora, each coxite of abdominal segments II–VII bearing only one pair of retractile vesicles, and a tertiary-type ovipositor [23,30].

Archaeognatha possesses a mitochondrial genome that exhibits structural conservation patterns commonly found in typical insect taxa. Characterized by matrilineal transmission, accelerated evolutionary dynamics, and non-recombining properties, this genomic system has proven to be a valuable tool for molecular studies aimed at testing and refining phylogenetic hypotheses [32,33,34]. Evidence indicates that intergenic regions can serve as homologous characters for genus-level classification and frequently form stem-loop or hairpin secondary structures [35,36]. These hairpin motifs are widely distributed in the intergenic spacers between mitochondrial PCGs of diverse metazoan taxa, including insects [37,38,39]. Guan et al. were the first to identify long hairpin structures located between the *ND1* and *16S rRNA* genes in the subfamilies Petrobiinae and Machilinae, revealing a strong correlation between their spatial arrangement and phylogenetic relationships [36]. These discoveries highlight the functional and evolutionary significance of mitochondrial genome architecture.

In recent years, research on phylogenetic relationships has expanded in both scope and analytical rigor. The majority of phylogenetic studies have consistently supported the monophyly of Hexapoda and Archaeognatha [2,40,41,42,43,44]. However, the internal classification system of Archaeognatha remains complex and continues to be a subject of active scientific debate. Ma et al. demonstrated that both Machilidae and its subfamily Machilinae are paraphyletic, whereas Petrobiellinae forms a monophyletic clade together with Meinertellidae. This finding challenges the previous morphological hypothesis of monophyly and provides critical molecular evidence for revising the phylogenetic framework of Archaeognatha [3]. Zhang et al. confirmed the monophyly of Meinertellidae through an integrative analysis of morphological and DNA data from 15 Burmese amber specimens [2]. Montagna subsequently expanded the analysis by incorporating two Triassic fossils and species from Zygentoma, further supporting for the monophyly of Meinertellidae and the paraphyly of the broadly defined Machilidae [45]. Cen et al. built upon previous work by including sequence data from six *Pedetontus* species, yielding preliminary support for the monophyly of this genus and revealing its sister-group relationship with *Pedetontinus* [46]. Palacios-Martinez et al. conducted a Bayesian phylogenetic analysis integrating *COI*, *18S rDNA*, and *ITS2* sequence data across multiple loci, demonstrating that Machilidae and its two subfamilies—Machilinae and Petrobiinae—are non-monophyletic [1]. Collectively, these findings underscore the need for an integrative taxonomic revision that incorporates multi-locus and multidisciplinary evidence to resolve systematic inconsistencies and clarify cladogenetic patterns within this lineage.

Fossil-calibrated molecular clocks have become a widely accepted and effective method for reconstructing the deep phylogeny of insects [33,34,46,47]. However, the evolutionary history of Archaeognatha remains incompletely resolved due to an incomplete and sparse fossil record [46]. While the earliest potential representatives of Archaeognatha are dated to the Devonian period [48,49], definitive crown-group fossils have been found exclusively in Late Carboniferous to Early Permian strata from France and North America, supporting the hypothesis that the order originated during the Late Carboniferous [50,51]. Molecular evidence from Misof et al. suggests that the divergence of Archaeognatha from other insect lineages began approximately 479 million years ago (Mya) [52]. Zhang et al. further demonstrated, through a comprehensive phylogenetic framework combining fossil morphology with biomolecular evidence from Burmese amber specimens, that the two major families within Archaeognatha diverged well before the Cretaceous [2]. Notably, Montagna inferred that the extant family Machilidae was already present during the Middle Triassic—approximately 100 million years earlier than previous estimates [45]. More recently, Cen et al. reported that Machilidae originated in the Middle Triassic (approximately 238.46 Mya), Meinertellidae diverged approximately 127.76 Mya during the Early Cretaceous, and the crown group of Archaeognatha emerged approximately 301.19 Mya during the Late Carboniferous, followed by a prolonged phase of diversification throughout the Mesozoic era [46]. Despite these advances, the evolutionary history of Archaeognatha remains incompletely resolved, underscoring the importance of integrative analyses that combine fossil evidence with molecular datasets to more accurately reconstruct its diversification dynamics.

Although the prevailing view holds that the mitochondrial genome evolves under neutral or nearly neutral conditions [53], accumulating evidence suggests that mitochondrial protein-coding genes (PCGs) associated with environmental adaptation exhibit signatures of significant positive selection [54,55,56]. Given their essential role in energy metabolism during responses to environmental stressors, mitochondrial genomes provide a critical framework for investigating mechanisms of positive selection and adaptive evolution, particularly under conditions of strong natural selection. Xu et al. conducted a positive selection analysis on Heptageniidae taxa inhabiting cold environments and identified 27 positively selected sites across eight protein-coding genes [56]. Feng et al. performed a similar analysis on *Chionea* in cold environments and detected significant signals of positive selection in the mitochondrial genes *COIII*, *ND6*, and *ND5*, suggesting that the positive selection of these three genes may represent a key adaptive mechanism enabling *Chionea* species to survive in low-temperature habitats [57]. Faddeeva et al. examined selective pressures across Hexapoda lineages and found that 250 genes exhibited significant positive selection, indicating a potential role for these genes in lineage divergence [58]. Collectively, these studies underscore the importance of comparative mitochondrial genomic analyses across diverse taxa for understanding the functional dynamics of positive selection in adaptation to extreme environments and in shaping evolutionary trajectories.

Building upon the current state of research, this study aims to achieve the following objectives: (1) to compare the mitochondrial genomes of 14 species representing the genera *Pedetontus* and *Pedetontinus*; (2) to reconstruct phylogenetic topology and estimate divergence times within Archaeognatha; and (3) to examine whether the mitochondrial genome of *Pd. silvestrii* from temperate region has undergone positive selection during cold adaptation and whether similar selection signals are present in *Pedetontus* species from the tropical region in response to high-temperature adaptation.

## 2. Materials and Methods

### 2.1. Specimen Acquisition and Genomic DNA Extraction

Detailed sample information is presented in Table 1. For simplicity, the generic name *Pedetontus* is abbreviated as *Pd.* and *Pedetontinus* is abbreviated as *Pn*. throughout the text. Following sample collection, morphological examination was conducted on fourteen specimens utilizing a Nikon SMZ-1500 stereoscopic microscope (Nikon, Tokyo, Japan). Based on diagnostic morphological characters from established taxonomic references [9,10,23], five species were tentatively assigned to the genus *Pedetontinus*, and nine species to the genus *Pedetontus*. After species identification, all tissue samples were preserved in absolute ethanol (−20 °C) and stored under controlled conditions at the Laboratory of Evolution and Molecular Ecology, Zhejiang Normal University. Genomic DNA was extracted from muscle tissues using the Ezup Column Animal Genomic DNA Purification Kit (Sangon Biotech, Shanghai, China), adhering strictly to the manufacturer’s operational guidelines.

### 2.2. Sequencing and Assembly of the Mitochondrial Genome

Polymerase chain reaction (PCR) amplification of the *COI* gene fragment was performed using the universal insect primers LCO1490 and HCO2198 following genomic DNA extraction [59]. Species identification was conducted through sequence alignment against the NCBI BLAST database (https://blast.ncbi.nlm.nih.gov/Blast.cgi, accessed on 30 April 2025). The complete mitochondrial genome was amplified using 13 pairs of species-specific primers designed by Zhang et al. [60], combined with Sanger sequencing technology. All PCR products were purified via gel electrophoresis prior to bidirectional sequencing (Sangon Biotech, Shanghai, China). Sequence assembly and verification were performed using the SeqMan Pro module within DNASTAR v6.0 software [61] to ensure high data accuracy.

### 2.3. Sequence Annotation and Analyses

tRNA identification and annotation were conducted using MITOS2 (https://usegalaxy.eu/, accessed on 15 May 2025) [62]. Comparative analyses of the *12S/16S rRNA* and 13 PCGs were conducted based on multiple sequence alignments generated by Clustal W [63,64]. Genetic distances were calculated utilizing the Kimura 2-parameter model in MEGA version 11 [65]. PhyloSuite v.1.2.3 [66] was utilized to compute AT content and assess relative synonymous codon usage across the fourteen mitochondrial genomes. The complete mitochondrial genomes (GenBank accession numbers: PX391406-PX391419) were visualized using CG View Server V1.0 (http://cgview.ca/, accessed on 18 May 2025) [67], and the resulting figures were subsequently refined with Adobe Illustrator 2021 [68]. RNA secondary structure prediction tools were employed to detect potential hairpin motifs (http://rna.tbi.univie.ac.at/, accessed on 20 May 2025) [69]. Nucleotide composition bias was quantified using standard deviation-based formulas: AT bias = (A − T)/(A + T) and GC bias = (G − C)/(G + C) [70].

### 2.4. Phylogenetic Analyses

To comprehensively elucidate the evolutionary relationships within Archaeognatha, this study integrated 14 newly sequenced mitochondrial genomes with 20 previously published mitochondrial genomes retrieved from the NCBI database [3,36,46,60,71,72,73,74,75], along with two mitochondrial genomes from Collembola [76] used as outgroups. The final dataset comprises a total of 36 species (Table S1), including one representative from Meinertellidae, 33 species from Machilidae and the selected outgroup taxa: *Podura aquatica* (NC_006075) and *Onychiurus orientalis* (NC_006074).

A standardized phylogenetic analysis pipeline was applied to the 13 PCGs using PhyloSuite v1.2.3 [66]. The workflow consisted of three main steps: (1) multiple sequence alignment using MAFFT v7 [77]; (2) trimming of highly variable regions using Gblocks v0.91b [78] to improve alignment accuracy; and (3) sequence assembly using integrated software tools [79].

Codon saturation was evaluated using DAMBE v7.3.11 [80], confirming the absence of substitution saturation, particularly at the third codon position. Phylogenetic reconstruction was conducted using both Bayesian inference (BI) and maximum likelihood (ML), based on nucleotide sequences from all three codon positions across the 13 PCGs [81,82]. Model selection was performed in PartitionFinder v2.2.1 [83] under the Bayesian Information Criterion (BIC) [84], resulting in an optimal data partitioning scheme and corresponding substitution models, as detailed in Table 2. Phylogenetic analyses was performed using two independent approaches: (1) maximum likelihood analysis implemented in IQ-TREE v2.0 [85], with 1000 ultrafast bootstrap replicates used to assess nodal support; and (2) Bayesian inference conducted in MrBayes v3.2 [86], involving 10 million generations of MCMC sampling, with the first 25% of samples discarded as burn-in to minimize initial state bias. The phylogenetic trees were visualized using FigTree v1.4 [87] and further refined in Adobe Illustrator [68] to enhance clarity and overall presentation quality.

### 2.5. Estimation of Divergence Times

The selection of fossil calibration points is a crucial step in divergence times estimation [88,89,90]. To establish robust temporal references, we integrated data from peer-reviewed literature and the Paleobiology Database (https://paleobiodb.org/, accessed on 16 June 2025). According to the insect phylogeny proposed by Misof et al. [52], the Archaeognatha-Collembola cladogenic split was dated to approximately 479 million years ago (Mya) during the Early Ordovician radiation, which was implemented as a prior constraint for the root node age in our analysis. Four fossil calibration points were selected for this study: (1) *Dasyleptus lucasi* Brongniart, 1885 (order Archaeognatha), dated to approximately 298.9–303.7 Mya [51]. (2) *Machilis acuminata* (family Machilidae) dated to approximately 33.9–38.0 Mya [91]. (3) *Gigamachilis triassicus* (family Machilidae), dated to approximately 235–242 Mya [45]. (4) *Cretaceomachilis libanensis* (family Meinertellidae), dated to approximately 125.45–130.00 Mya [92]. Divergence times were estimated using MCMCTree within the PAML v4.8 software package [93], based on the BI phylogenetic tree topology. First, the nucleotide substitution rate was calculated using Baseml, and the gradient of branch lengths along with the associated Hessian matrix was obtained under a Bayesian framework. Subsequently, species divergence times were inferred using the approximate likelihood calculation method (usedata = 2). In the MCMCTree analysis, the following parameters were applied: the burn-in period of 1,000,000 iterations, with sampling every 1000 generations, resulting in the collection of 100,000 effective samples for downstream analyses. Optimization of MCMC chain convergence and mixing was verified using Tracer v1.7.1 [94]. All model parameters were assessed for effective sample size (ESS), showing that ESS values exceeded the recommended threshold of 200 for each parameter, confirming sufficient sampling efficiency and reliability of the estimates [95]. Estimated divergence time estimates across phylogenetic branches were visualized using FigTree v1.4 [87], and the resulting figures were further refined in Adobe Illustrator [68] to improve clarity and presentation quality.

### 2.6. Selection Pressure Analyses

The EasyCodeML v1.41 software [96] was employed to assess selective pressures acting on PCGs within the mitochondrial genomes of Archaeognatha. To investigate molecular evolutionary patterns of PCGs under different thermal regimes, populations of *Pd. silvestrii* from temperate regions (In proximity to 40° N), characterized by average winter temperatures below 0 °C, were designated as foreground branches representing the temperate group. In contrast, *Pd. hainanensis* and *Pd. bawanglingensis* from tropical regions (near 19° N), which experience average summer temperatures exceed 30 °C, were assigned as foreground branches representing the tropical regions group. Selection pressure analyses were carried out using three types of codon models—branch models, branch-site models, and clade models—to detect signatures of selection on specific lineages and sites. The branch model is employed to identify lineage-specific variations in evolutionary rates [97]. The branch-site model enables comparative analysis between a null model (Model Anull), which assumes only neutral and purifying selection, and an alternative model (Model A), thereby enabling the detection of positive selection on predefined foreground branches [98]. The clade model framework facilitates the identification of divergent selection pressures across distinct evolutionary lineages [99]. Additionally, a site model was employed to evaluate overall selective pressures without specifying foreground branches [100]. Model comparisons were conducted using likelihood ratio tests (LRTs) [101]. For statistical validation of adaptive evolution signals, the Bayesian empirical Bayes (BEB) approach was applied to estimate posterior probabilities at individual sites, with a threshold of ≥0.95 used to ensure reliable inference [102].

## 3. Results

### 3.1. Composition of Mitogenomes

All 14 mitochondrial genomes possess a double-stranded DNA structure, ranging in size from 14,625 bp (*Pn. jinzhaiensis*) to 15,808 bp (*Pd. bawanglingensis*) (Table S1; Figure S1). The control region could not be amplified in *Pn. jinzhaiensis*, and both the control region and *trnI* were not successfully amplified in *Pd. formosa*. Mitochondrial gene lengths, AT content and other relevant information are summarized in Table S2. The initiation and termination codons for each species are detailed in Table S3. The initiation codons are predominantly of the ATN type, however, GTG is also utilized as an initiation codon in several cases, such as in *COII* and *ND2*. With regard to stop codons, most PCGs utilize canonical termination signals (TAA or TAG). Incomplete stop codons (T or TA) were detected in *COI*, *COII*, *COIII*, *ND5*, and *ND4*. Relative synonymous codon usage (RSCU) is shown in Figure S2, and the corresponding codon usage frequencies are provided in Table S4. Codon usage analysis reveals that UUU, AUU, AUA, and UUA are the most frequently used codons, each occurring more than 200 times. In contrast, codons with G or C at the third nucleotide position exhibit lower usage frequencies. Notably, Long hairpin structures were identified between the *ND1* and *16S rRNA* genes across the mitochondrial genomes of 14 species within *Pedetontus* and *Pedetontinus*. These structures range in length from 42 to 76 bp, with an average of approximately 60 bp (Figure S3).

### 3.2. Genetic and Intergroup Genetic Distance Analyses

Genetic distances between *Pedetontinus* and *Pedetontus* species, based on the *COI* gene, are summarized in Table S5. Overall genetic divergences range from 1.5% to 23%, with a mean value of 16.98%. Intraspecific genetic divergence within *Pedetontinus* ranges from 7.4% to 14.2%. However, genetic divergence within *Pedetontus* is comparatively higher. Genetic divergences between *Pedetontus* from the southern China and those from the Northeast region all exceed 15%. The pairwise genetic distances between *Pd. formosa* and *Pd. zhoui* (collected from Taiwan and Fujian, respectively) and *Pedetontus* from Zhejiang (excluding *Pd. cixiensis*) ranged from 11.5% to 13.1%. Genetic divergences between *Pd. hainanensis* and other *Pedetontus* species are all consistently high, exceeding 20%. Similarly, genetic distances between *Pd. bawanglingensis* and other *Pedetontus* species also exceed 20%. Furthermore, the genetic divergence between *Pd. hainanensis* and *Pd. bawanglingensis* is 16.1%. Additionally, genetic distances between *Pd. cixiensis* and other *Pedetontus* species range from 17.2% to 21.5%.

Based on the genetic distance analysis, *Pd. cixiensis*, *Pd. hainanensis*, and *Pd. bawanglingensis* exhibited substantial genetic divergence from other species within the same genus. To further assess the intergeneric variation, a stratified genetic distance analysis was conducted. Taxa were reclassified into distinct groups based on two criteria: genus-level taxonomy and geographical distribution. Specifically, species belonging to the same genus and originating from the same geographic region were assigned to the same group, whereas those from different genera or different geographical regions were placed in separate groups. The grouping scheme is as follows: Group 1 included five samples of the genus *Pedetontinus* and its reference sequence (KJ754502); Group 2 consisted of *Pd. hainanensis* and *Pd. bawanglingensis*; Group 3 comprised *Pd. cixiensis*; Group 4 included six samples of *Pedetontus* from southern regions of China (excluding *Pd. cixiensis*, *Pd. hainanensis* and *Pd. bawanglingensis*) along with its reference sequence (NC_051491); and Group 5 contained six samples of *Pedetontus silvestrii* from northeast China and its reference sequence (NC_011717). Genetic distances among these groups were subsequently calculated, and the results are summarized in Table S6.

Following the inter-group genetic distance analysis of the five defined groups, the samples were further categorized into two major genera: *Pedetontus* and *Pedetontinus*, and the inter-generic genetic distance between these two genera was calculated. The results revealed that the genetic distance between Group 2 (*Pd. hainanensis* and *Pd. bawanglingensis*) and Group 3, 4 and 5 (other *Pedetontus* groups) all exceeded 20%. In contrast, the genetic divergence between the genera *Pedetontus* (excluding *Pd. hainanensis* and *Pd. bawanglingensis*) and *Pedetontinus* was 19.8%.

### 3.3. Phylogenetic Analyses of Archaeognatha

**Figure 1:**
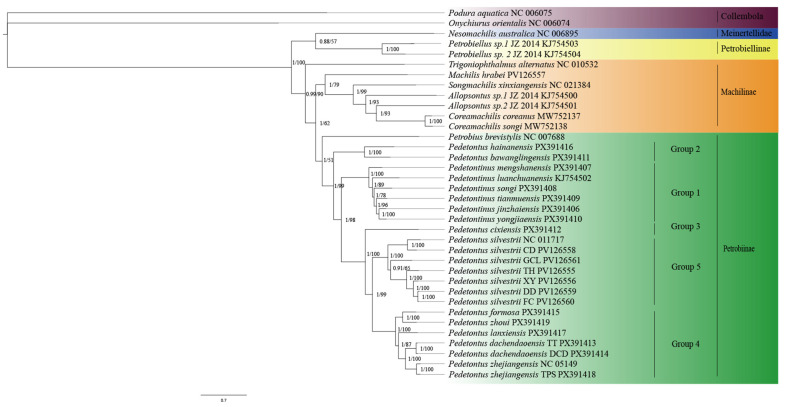
Phylogenetic relationships within Archaeognatha inferred through the analysis of 13 mitochondrial PCGs using ML and BI methods. Bootstrap values on the right side correspond to each branch, with posterior probabilities on the left.

Phylogenetic reconstruction was conducted using both BI and ML approaches, yielding congruent topological structures (Figure 1). The monophyly of Archaeognatha was strongly supported. However, the phylogenetic analyses did not support the monophyly of the family Machilidae, as species previously assigned to the subfamily Petrobiellinae were recovered within Meinertellidae and formed a closely related sister group to this family. Furthermore, the monophyly of the subfamily Machilinae could not be confirmed, as *Trigoniophthalmus alternatus*, a species taxonomically classified within Machilinae, was positioned outside the clade containing other representative species from the same subfamily.

The phylogenetic tree generated in this study reveals that the genus *Pedetontinus* included in this analysis forms a robustly supported monophyletic clade, whereas the genus *Pedetontus* sampled here is not monophyly. Specifically, all sampled individuals of *Pd. silvestrii* form a distinct and well-supported clade, and the majority of *Pedetontus* specimens collected from Zhejiang (excluding *Pd. cixiensis*) also cluster within a separate monophyletic lineage. *Pd. formosa* and *Pd. zhoui* from South China formed a distinct clade that is clearly separated from the Zhejiang-derived *Pedetontus* samples, representing an independent evolutionary lineage. Phylogenetic analysis indicates that *Pd. formosa* and *Pd. zhoui* form a sister group to most of the *Pedetontus* samples from Zhejiang (except *Pd. cixiensis*), while *Pd. silvestrii* is recovered as the sister lineage to this clade. Furthermore, *Pd. hainanensis* and *Pd. bawanglingensis* are resolved outside the main clade comprising *Pedetontus* and *Pedetontinus*, occupying a relatively basal position, suggesting that these two taxa may represent early-diverging lineages within the subfamily.

### 3.4. Divergence Time Calculation

**Figure 2:**
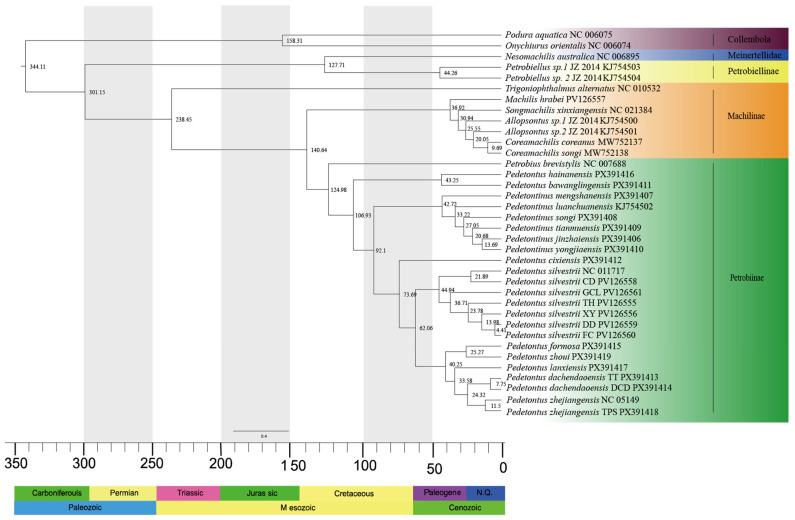
Time-calibrated phylogenetic tree of Archaeognatha inferred using the MCMCTree algorithm. Median divergence times are indicated at corresponding nodes. Stratigraphic age data are presented along the lower margin of the figure.

The chronogram, inferred from the BI phylogenetic tree, is presented in Figure 2. Phylogenetic analysis of Archaeognatha based on 13 mitochondrial PCGs reveals that the order originated during the Late Carboniferous period (301.15 Mya, 95% HPD: 298.88–303.67 Mya). *Trigoniophthalmus alternatus*, a member of the subfamily Machilinae, represents the earliest divergence from the main lineage, occurring in the Middle Triassic period (238.45 Mya, 95% HPD: 235–241.99 Mya). The split between the subfamilies Machilinae and Petrobiinae took place during the Jurassic period (140.64 Mya, 95% HPD: 94.65–222.39 Mya). *Pedetontus hainanensis* and *Pd. bawanglingensis* diverged from the main clade comprising the genera *Pedetontus* and *Pedetontinus* during the Early Cretaceous (106.93 Mya, 95% HPD: 70.46–170.92 Mya). The divergence between the genera *Pedetontus* and *Pedetontinus* occurred in the same geological period (92.1 Mya, 95% HPD: 60.61–145.97 Mya). *Pedetontus cixiensis* separated from the remaining members of the genus *Pedetontus* during the Late Cretaceous (73.69 Mya, 95% HPD: 46.24–114.85 Mya), followed by the divergence of *Pd. silvestrii* and *Pedetontus* populations from Zhejiang, Fujian, and Taiwan within the same epoch (62.06 Mya, 95% HPD: 38.68–98.34 Mya).

Moreover, our findings indicate that *Pedetontus* diverged earlier than *Pedetontinus*. The most recent common ancestor (MRCA) of *Pedetontus* originated during the Late Cretaceous (73.69 Mya, 95% HPD: 46.24–114.85 Mya), whereas the MRCA of *Pedetontinus* appeared in the Eocene (42.72 Mya, 95% HPD: 25.7–81.83 Mya). Divergence time analysis revealed three major diversification events during the Eocene: the origin of the MRCA of *Pedetontus silvestrii* at 44.94 Mya (95% HPD: 27.94–77.06 Mya), the divergence of the MRCA between the lineage comprising South China (*Pd. formosa* and *Pd. zhoui*) and Zhejiang populations (*Pd. lanxiensis*, *Pd. dachendaoensis* TT, *Pd*. *dachendaoensis* DCD, *Pd. zhejiangensis* TPS and *Pd. zhejiangensis* NC 051491) at 40.25 Mya (95% HPD: 26.17–69.29 Mya), and the emergence of the MRCA of *Pedetontus* from Zhejiang at 33.58 Mya (95% HPD: 21.28–58.81 Mya). The MRCA of *Pd. formosa* and *Pd. zhoui* diverged later during the Oligocene (25.27 Mya, 95% HPD: 11.89–46.44 Mya).

### 3.5. Positive Selection Analysis

The branch-site model was utilized to investigate selective pressures operating at the amino acid sites, based on aligned sequence data from 13 PCGs derived from 34 Archaeognatha species. When *Pedetontus silvestrii* were designated as the foreground branch, two amino acid sites were identified as potentially under positive selection, with statistically significant support (*p* < 0.001, BEB value ≥ 0.95), located at the 66th position of the *Cytb* gene and the 34th position of *ATP6* (Table 3). When *Pd. hainanensis* and *Pd. bawanglingensis* were used as foreground branches, six amino acid sites were found to be under positive selection, with statistically significant support (*p* < 0.001, BEB value ≥ 0.95), located at the 622nd position of the *Cytb* gene, the 499th position of *ATP6*, and the 623rd, 873rd, 1106th, and 1141st positions of *COI* (Table 4). Nevertheless, no significant signals of positive selection were detected using the branch model, site model, or evolutionary distinct branch model.

## 4. Discussion

### 4.1. Special Structure of Mitogenomes from Pedetontus and Pedetontinus

Analysis revealed a long hairpin structure located between the *ND1* and *16S rRNA* genes across the 14 mitochondrial genomes of *Pedetontus* and *Pedetontinus*, which is consistent with the findings reported by Guan et al. [36]. Notably, such long hairpin structures have so far been observed exclusively in species belonging to the subfamilies Petrobiinae and Machilinae, but not in those within Petrobiellinae or Meinertellidae [36]. Therefore, we propose that this structural feature may be phylogenetically informative and could represent a shared derived character (synapomorphy) for Petrobiinae and Machilinae. However, its precise biological function and the underlying mechanism of formation remain unclear and require further investigation.

### 4.2. Analysis of Phylogenetic Tree Topology

Our phylogenetic reconstruction reveals the non-monophyly of Machilidae, challenging the morphological classification system that has been widely used for this taxon [31]. This incongruence stems from the closer phylogenetic relationship between the subfamily Petrobiellinae and the family Meinertellidae, rather than their affinities with other groups within Machilidae—a pattern consistent with findings from previous phylogenetic studies [3,36,46]. However, the interpretation of Petrobiellinae’s phylogenetic position requires careful consideration of existing taxonomic uncertainties. As critically evaluated by Kaplin [103] and Mtow and Machida [104], the sequences of *Petrobiellus* used in our study and in previous analyses are taxonomically problematic. The sequences generated by Ma et al. may represent misidentified members of Meinertellidae rather than true *Petrobiellus* [3]. Therefore, while our analysis recovers the non-monophyly of Machilidae, the precise phylogenetic placement of Petrobiellinae should be interpreted not as a definitive conclusion but as a provisional hypothesis awaiting validation through future studies based on taxonomically verified voucher specimens.

Notably, *Petrobiellus* from Petrobiellinae exhibits morphological similarities to *Petrobius* from Petrobiinae, particularly in the underdeveloped distal jaw teeth with fused dental boundaries [4]. These morphological parallels may reflect either shared ancestry or convergent evolution [2,3]. However, molecular phylogenetic analyses indicate that these taxa are placed in distinct clades, with the two *Petrobiellus* species forming a monophyletic clade together with *Nesomachilis australica* from the family Meinertellidae. This finding, consistent with the observations of Kaplin [103] and Mtow and Machida [104], further suggests that there may be potential taxonomic inconsistencies or misidentifications within *Petrobiellus*, which warrant further investigation.

Although the monophyly of Meinertellidae has been supported by the studies of Zhang et al. and Montagna [2,45], only a single complete mitochondrial genome sequence from this family is currently available in the NCBI database [72]. Consequently, the present study included only one representative species from Meinertellidae, which limits the ability to comprehensively assess and validate the monophyletic status of this taxon. Nevertheless, several morphological characteristics provide evidence in support of the monophyly of Meinertellidae, including the absence of antennal scales, the lack of accessory reproductive structures, and the reduction in the size of abdominal plates [1].

Phylogenetic analyses further support the paraphyly of Machilinae, as evidenced by the placement of *T. alternatus* within Petrobiinae rather than among other Machilinae species. This finding aligns with the results reported by Guan et al. and Ma et al. [3,36]. Additionally, it has been demonstrated that the traditional classification of *Trigoniophthalmus*, which relies on ancestral morphological traits, has limited taxonomic accuracy and leads to inconsistencies between phylogenetic and morphological classifications, as previously noted by Guan et al. [36]. Therefore, we recommend that the classification of the genus *Trigoniophthalmus* be reevaluated using an integrative approach that combines phylogenetic data with morphological evidence.

Within the phylogenetic framework, *Pedetontinus* as sampled here is robustly supported as a monophyletic group, in contrast to the paraphyletic structure observed in *Pedetontus* as represented here. This non-monophyletic pattern conflicts with the results of previous phylogenetic studies by Shen et al. and Cen et al., which inferred a monophyletic origin for *Pedetontus* [46,75]. We propose that this discrepancy may stem from the substantially expanded taxon sampling and broader geographical representation in our study, which includes specimens from multiple regions across southern, eastern, northern, and northeastern China. With regard to *Pd. silvestrii*, the sampling methodology employed in this study was consistent with that of Cen et al., and the topology of the reconstructed phylogenetic tree showed a high level of agreement with their results [46]. Notably, *Pd. cixiensis* collected from Cixi, Zhejiang Province, does not group within other specimens from Zhejiang but instead diverges outside the entire *Pedetontus* crown group, forming a distinct and elongated branch. Furthermore, the genetic distance between the *COI* gene of *Pd. cixiensis* and those of other *Pedetontus* specimens from Zhejiang exceeds 17%, indicating that this lineage may represent an early divergence event, which merits further investigation.

### 4.3. Divergence Time Estimation and Evolutionary Node Calibration

Divergence time estimation indicates that the family Machilidae originated during the Middle Triassic period (238.45 Mya, 95% HPD: 235–241.99 Mya), a result consistent with the findings reported by Montagna [45]. The divergence between the family Meinertellidae and the subfamily Petrobiellinae occurred during the Early Cretaceous (127.71 Mya, 95% HPD: 125.45–130 Mya), a timeframe that aligns with the fossil record of *Cretaceomachilis libanensis*, discovered in Early Cretaceous strata of Lebanon [92]. The earlier divergence of Machilidae relative to Meinertellidae is supported by morphological features, including the ovipositor, aedeagus, paragenitalia, coxal sac, and tarsus [2,3]. Moreover, the divergence time tree constructed by Cen et al. also supports this temporal pattern of diversification [46]. According to our analysis, the most recent common ancestor (MRCA) of Machilidae and Meinertellidae dates to the Early Cretaceous (127.71 Mya, 95% HPD: 125.45–130 Mya), which is notably later than previous estimates that placed this divergence at the Jurassic-Cretaceous boundary (approximately 146 Mya) [45,52]. We hypothesize that this discrepancy may stem from insufficient sampling of Meinertellidae in prior analysis, which could have affected the accuracy of the estimated divergence times. Inferred via divergence dating, the subfamilies Machilinae, Petrobiinae, and Petrobiellinae diverged sequentially during the Middle Triassic (approximately 238.45 Mya), Early Cretaceous (approximately 124.98 Mya), and Eocene (approximately 44.26 Mya), respectively. This temporal divergence pattern corresponds to the framework previously proposed by Cen et al. [46]. Notably, the estimated divergence time of the genus *Machilis* during the Eocene (36.92 Mya, 95% HPD: 34.33–38.14 Mya) is in close agreement with the fossil record of this genus preserved in Baltic Eocene amber [105].

The Mesozoic Era, a critical interval in Earth’s evolutionary history, was characterized by major environmental changes that played a fundamental role in shaping modern insect communities [106]. Evidence indicates that the substantial rise in atmospheric carbon dioxide levels during the Cretaceous period maintained a prolonged warm climate. Together with the plate tectonic movements driven by the rifting of Gondwana, these factors synergistically promoted the reorganization of global vegetation distribution patterns [107,108]. Against this climatic and geological background, the adaptive radiation of bryophytes emerged as a pivotal event in ecological evolution during the Cretaceous. Molecular phylogenetic studies indicate that epiphytic moss lineages underwent a phase of rapid diversification, giving rise to a broad spectrum of adaptive strategies [109]. The intensification of plant-insect coevolutionary interactions during this period further facilitated the diversification of multiple insect groups, including the genus-level differentiation within the order Archaeognatha. During the Cretaceous-Paleogene transition, the rapid diversification of numerous plant, animal, and fungal lineages triggered a major ecological transformation known as the “Cretaceous Terrestrial Revolution”, which drove the evolutionary progression toward more complex and diverse insect-plant symbiotic systems [110]. Our phylogenetic analysis reveals that *Pedetontus* and *Pedetontinus* diverged from a common ancestral lineage in the late Mesozoic, a timeframe that closely aligns with a major phase of reorganization in the global biogeographic patterns of bryophytes [109]. Collectively, these findings highlight the profound impact of Mesozoic environmental changes on the evolutionary assembly of contemporary insect communities.

The MRCA of *Pd. formosa* and *Pd. zhoui* diverged during the late Oligocene (25.27 Mya, 95% HPD: 11.89–46.44 Mya). This molecular dating estimate appears inconsistent with the geological timeline of Taiwan Island’s formation. Geological studies indicate that the fundamental topographic framework of Taiwan Island was established approximately 6.5 Mya, resulting from oblique plate interactions along the Luzon-Eurasian tectonic boundary [111]. Prior to this island formation, however, the region underwent significant tectonic evolution: continental rifting occurred during the Paleogene (39–50 Mya), followed by the opening of the South China Sea during the Oligocene to Middle Miocene (16–33 Mya) [112]. These tectonic processes likely contributed to the opening of the Taiwan Strait and induced localized crustal movements, which may have influenced biogeographic patterns and speciation events. Such dynamic geological settings could have provided diverse ecological niches for the ancestral populations of *Pd. formosa* and *Pd. zhoui*, thereby facilitating their divergence and adaptive evolutionary trajectories.

### 4.4. Genetic-Morphological Discrepancy in Pedetontus Taxonomy

Morphological methods have long served as the primary tools for species identification. Nevertheless, evolutionary relationships inferred from these approaches often exhibit substantial inconsistencies when compared with genetic evidence [113,114,115]. Due to the inherent limitations of traditional morphological identification systems in achieving accurate taxonomic classification, there is an increasing need for integrative frameworks that enable the systematic identification, refinement, and validation of diagnostic traits with improved discriminatory power. Phylogenetic and genetic distance analyses of *Pedetontus* have uncovered substantial taxonomic discrepancies within the genus. Comparative data indicate that the intergeneric genetic distance between *Pedetontus* and *Pedetontinus* was 19.7%, whereas the interspecific genetic distances between Group 2 (*Pd. hainanensis* and *Pd. bawanglingensis*) and other conspecific species exceeded 20%, ranging from 20.2% to 23.0%. Notably, the level of intrageneric genetic differentiation exceeds that typically observed between genera, providing strong support the hypothesis of a potential misclassification of *Pd. hainanensis* and *Pd. bawanglingensis* at the generic level. Phylogenetic reconstruction corroborates this conclusion, showing their phylogenetic divergence from congeneric *Pedetontus* species. Divergence time estimation further indicates that the lineage containing the Hainan population diverged approximately 106.93 Mya, significantly earlier than the divergence times of other *Pedetontus* species. Based on the molecular phylogenetic findings presented above, we propose an integrative taxonomic revision of *Pedetontus* to identify key morphological diagnostic characters suitable for genus-level classification. Furthermore, we recommend the development of a standardized and operationally robust framework for species identification that integrates morphological, molecular phylogenetic, and biogeographic evidence.

### 4.5. Positive Selection Pressure Analysis

Branch-site model analysis identified one site under positive selection in *Cytb* and an additional site in *ATP6* when *Pd. silvestrii* was designated as the foreground branch. *Cytb*, recognized as the major transmembrane component of complex III, is essential for ATP production [116]. As a core subunit of the mitochondrial respiratory chain, *Cytb* primarily mediates electron transfer from the substrate side to cytochrome *c*1. Its specialized energy-transducing form, cytochrome *b*T, is critically involved in coupling electron transport with proton gradient formation through the inner mitochondrial membrane, thereby serving as a key functional unit in oxidative phosphorylation and cellular energy conservation [117]. ATP synthase is also a core component of the mitochondrial respiratory chain, utilizes the proton motive force across the inner mitochondrial membrane to catalyze ATP synthesis from ADP and inorganic phosphate [118]. As a critical subunit of the enzyme, *ATP6* plays an essential role in oxidative phosphorylation [119].

Additionally, branch-site model analysis with *Pd. hainanensis* and *Pd. bawanglingensis* designated as foreground branches identified six positively selected sites: four in *COI*, one in *ATP6*, and one in *Cytb*. Complex IV catalyzes the final step of the electron transport chain by transferring electrons from reduced cytochrome c to oxygen, thereby reducing oxygen and generating a proton gradient [120]. The *COI* subunit is essential for initiating complex IV assembly and constitutes one of its catalytic core components [120,121].

It is noteworthy that in the analysis of positive selection sites under two distinct environmental conditions, both the *Cytb* and *ATP6* genes were identified. This finding indicates that these genes may have been subject to natural selection during both temperate and tropical environmental adaptation processes, suggesting their potential role in a shared adaptive mechanism in response to temperature-related selective pressures. In studies investigating other species’ responses to temperate environments, mitochondrial genes such as *Cytb* and *ATP6* have exhibited significant signals of positive selection. Xu et al. identified 27 positively selected sites in Heptageniidae species in their study on cold adaptation, including two sites in the *Cytb* gene [56]. Hong et al. reported five amino acid sites under positive selection in Hylidae species inhabiting cold environments, including residues within the *Cytb* gene [122]. Ngatia et al. demonstrated evidence of positive selection acting on six genes—including *Cytb* and *ATP6*—in *Mammuthus primigenius* under cold stress conditions [123]. Collectively, these findings demonstrate that key mitochondrial genes associated with energy metabolism have undergone adaptive evolution during biological responses to low-temperature environments. Notably, while existing studies on positive selection have predominantly focused on temperate species, the present investigation into the adaptive evolutionary patterns of tropical species provides novel insights.

Overall, the PCGs of *Pedetontus* are functionally associated with energy metabolism and are subject to positive selection, potentially reflecting adaptive mechanisms that enable the species to meet energy demands and maintain normal physiological functions under temperate and tropical environmental stress.

## 5. Conclusions

This study involved the analysis of mitogenomes from 14 species representing the genera *Pedetontus* and *Pedetontinus*. Phylogenetic assessments employing ML and BI methodologies yielded the following findings: (1) the family Machilidae is non-monophyletic; (2) the subfamily Petrobiellinae and the family Meinertellidae form a single clade, and the subfamily Machilinae is also non-monophyletic; (3) within the subfamily Petrobiinae, *Pedetontinus* is monophyletic, whereas *Pedetontus* is non-monophyletic, with *Pd. hainanensis* and *Pd. bawanglingensis* diverging early from the main lineage. Divergence time estimation indicates that the diversification of Petrobiinae occurred during the Mesozoic, leading to the emergence of the genera *Pedetontus* and *Pedetontinus*, a process potentially driven by the co-evolution between organisms and their environment. By integrating analyses of genetic distance, phylogenetic topology, and estimations of divergence timing, we propose a re-evaluation of the species status of *Pd. hainanensis* and *Pd. bawanglingensis*. Future research should aim to identify genus-level morphological diagnostic characters to refine the current taxonomic framework. Selection pressure analysis reveals that the mitochondrial PCGs of *Pedetontus* have undergone positive selection under both temperate and tropical environmental stress, likely reflecting adaptive responses to altered energy demands.

## Figures and Tables

**Table 1:** Detailed Sample Information for the Fourteen Specimens.

Species	Sampling Localities	Geographic Coordinates	**Accession No.**
*Pedetontinus songi*	Taimushan, Fujian	27°07′27″ N, 120°11′05″ E	PX391408
*Pedetontinus jinzhaiensis*	Jinzhai, Anhui	31°18′13″ N, 115°40′38″ E	PX391406
*Pedetontinus mengshanensis*	Mengshan, Shandong	35°31′07″ N, 117°49′17″ E	PX391407
*Pedetontinus tianmuensis*	Chuzhou, Anhui	32°20′42″ N, 118°25′30″ E	PX391409
*Pedetontinus yongjiaensis*	Yongjia, Zhejiang	28°09′14″ N, 120°41′29″ E	PX391410
*Pedetontus lanxiensis*	Lanxi, Zhejiang	29°12′33″ N, 119°27′38″ E	PX391417
*Pedetontus hainanensis*	Wuzhishan, Hainan	18°51′43″ N, 109°37′33″ E	PX391416
*Pedetontus formosa*	Baxiandong, Taiwan	23°23′28″ N, 121°29′04″ E	PX391415
*Pedetontus zhoui*	Taimushan, Fujian	27°07′27″ N, 120°11′05″ E	PX391419
*Pedetontus bawanglingensis*	Bawangling, Hainan	19°03′49″ N, 109°10′14″ E	PX391411
*Pedetontus dachendaoensis* DCD	Dachendao, Zhejiang	28°28′20″ N, 121°54′06″ E	PX391414
*Pedetontus cixiensis*	Cixi, Zhejiang	30°10′18″ N, 121°15′39″ E	PX391412
*Pedetontus dachendaoensis* TT	Taizhou, Zhejiang	28°50′49″ N, 121°06′50″ E	PX391413
*Pedetontus zhejiangensis* TPS	Suzhou, Zhejiang	31°01′59″ N, 120°51′14″ E	PX391418

**Table 2:** The best partitioning strategy for phylogenetic analyses, along with the associated alternative models, detailing the model names and the site classifications allocated to each partition.

Subset	Best Model	Partition Names
Partition_1	GTR + I + G	COII_codon1, COIII_codon1, ND3_codon1, Cytb_codon1, ATP6_codon1
Partition_2	GTR + I + G	COI_codon2, COIII_codon2, Cytb_codon2, ATP6_codon2, COII_codon2
Partition_3	GTR + G	ND3_codon3, COIII_codon3, Cytb_codon3, COI_codon3, COII_codon3
Partition_4	TIM + G	ND6_codon3, ND2_codon3, ATP8_codon3, ATP6_codon3
Partition_5	GTR + I + G	ND6_codon1, ATP8_codon2, ND2_codon1, ATP8_codon1
Partition_6	SYM + I + G	COI_codon1
Partition_7	GTR + I + G	ND5_codon1, ND4_codon1, ND1_codon1, ND4L_codon1
Partition_8	GTR + I + G	ND1_codon2, ND5_codon2, ND4_codon2, ND4L_codon2
Partition_9	TVM + G	ND5_codon3, ND4L_codon3, ND1_codon3, ND4_codon3
Partition_10	TVM + I + G	ND6_codon2, ND2_codon2, ND3_codon2

**Table 3:** Codon-based branch site modeling identified adaptive evolution signals by assigning *Pedetontus silvestrii* as the foreground lineage and utilizing remaining Archaeognatha species as reference clades. The symbol “*” indicates that the posterior probability for this site in the BEB analysis is ≥0.95, providing strong statistical evidence that the site may be under positive selection.

Branch Site Model (BSM)										
Model	np	Ln L	Estimates of Parameters					Model Compared	LRT *p*-Value	Positive Sites
Model A	71	−221,646.437857	Site class	0	1	2a	2b	Model A vs. Model A null	0.000884974	66 S 0.965 *, 1168 S 0.980 *
			Proportion	0.83698	0.15409	0.00754	0.00139			
			Background ω	0.05727	1.00000	0.05727	1.00000			
			Foreground ω	0.05727	1.00000	46.18276	46.18276			
Model A null	70	−221,651.964843								

**Table 4:** Codon-based branch site modeling identified adaptive evolution signals by assigning *Pedetontus hainanensis* and *Pd. bawanglingensis* as the foreground lineage and utilizing remaining Archaeognatha species as reference clades. The symbol “*” indicates that the posterior probability for this site in the BEB analysis is ≥0.95, providing strong statistical evidence that the site may be under positive selection.

Branch Site Model (BSM)										
Model	np	Ln L	Estimates of Parameters					Model Compared	LRT *p*-Value	Positive Sites
Model A	71	−221,635.143815	Site class	0	1	2a	2b	Model A vs. Model A null	0.000018027	622 F 0.953 *, 1633 Q 0.967 *, 2591 S 0.968 *, 2841 V 0.960 *, 3075 I 0.955 *, 3109 M 0.963 *
			Proportion	0.83368	0.15291	0.01133	0.00208			
			Background ω	0.05721	1.00000	0.05721	1.00000			
			Foreground ω	0.05721	1.00000	106.37636	106.37636			
Model A null	70	−221,644.337408								

## Data Availability

Data to support this study are available from the National Center for Biotechnology Information (https://www.ncbi.nlm.nih.gov) (accessed on 25 July 2025). The GenBank numbers are PX391406-PX391419.

## Supplementary Materials

The following supporting information can be downloaded at: https://www.mdpi.com/article/10.3390/insects16121194/s1, Figure S1: Circular genome maps illustrate the mitochondrial genomes of fourteen specimens representing the genera *Pedetontus* and *Pedetontinus*. Figure S2: Relative Synonymous Codon Usage (RSCU) patterns in 14 mitochondrial genomes. Figure S3: Inferred hairpin structures of 14 mitogenomes. The circle means the length between the *NDI* gene and *16S RNA* gene. Table S1: Species data employed in the construction of the phylogenetic tree. Table S2: The lengths of mitochondrial genes, AT content, AT skew and GC skew of 14 mitochondrial genomes. Table S3: Location of features of fourteen mitogenomes. Table S4: Comparative analysis of codon usage frequency profiles among 14 mitochondrial genomes. Table S5: Genetic distance analysis of the COI gene among species of *Pedetontinus* and *Pedetontus* is included in this study. Table S6: Pairwise genetic distance values among *Pedetontus* and *Pedetontinus* groups.
