# Supplementary material for: Comparative Mitogenomics of Pedetontus and Pedetontinus (Insecta: Archaeognatha) Unveils Phylogeny, Divergence History, and Adaptive Evolution
Source: Insects. 2025 Nov 24;16(12):1194. doi: 10.3390/insects16121194 (PMC12733737; doi:10.3390/insects16121194)
Supplement: Supplementary file 1 [file insects-16-01194-s001.zip › Figure S1 Circular genome maps illustrate the mitochondrial genomes of fourteen specimens representing the genera Pedetontus and Pedetontinus..pdf]

Figure S1. Circular genome maps illustrate the mitochondrial genomes of fourteen specimens representing the genera *Pedetontus* and *Pedetontinus*.

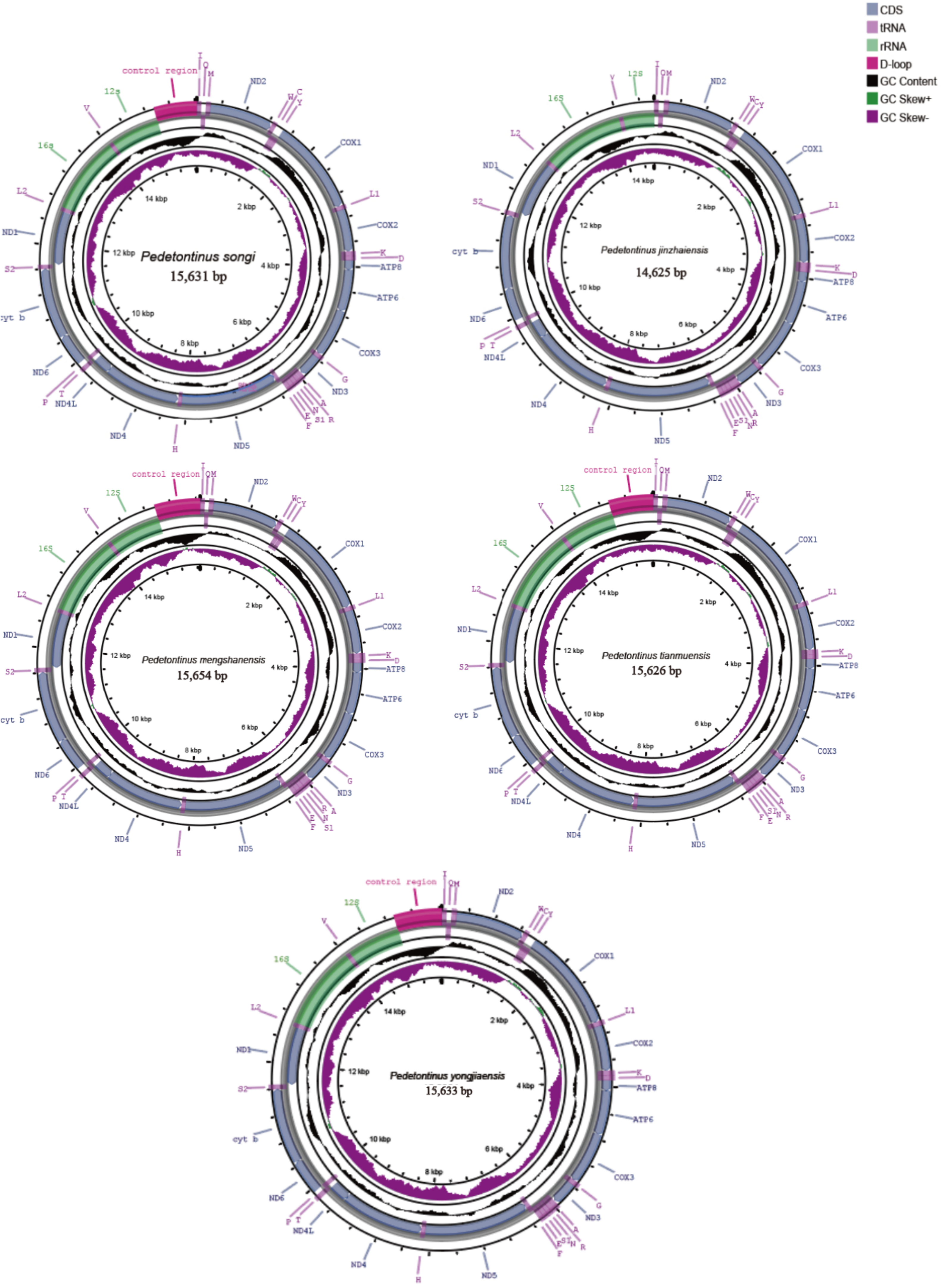

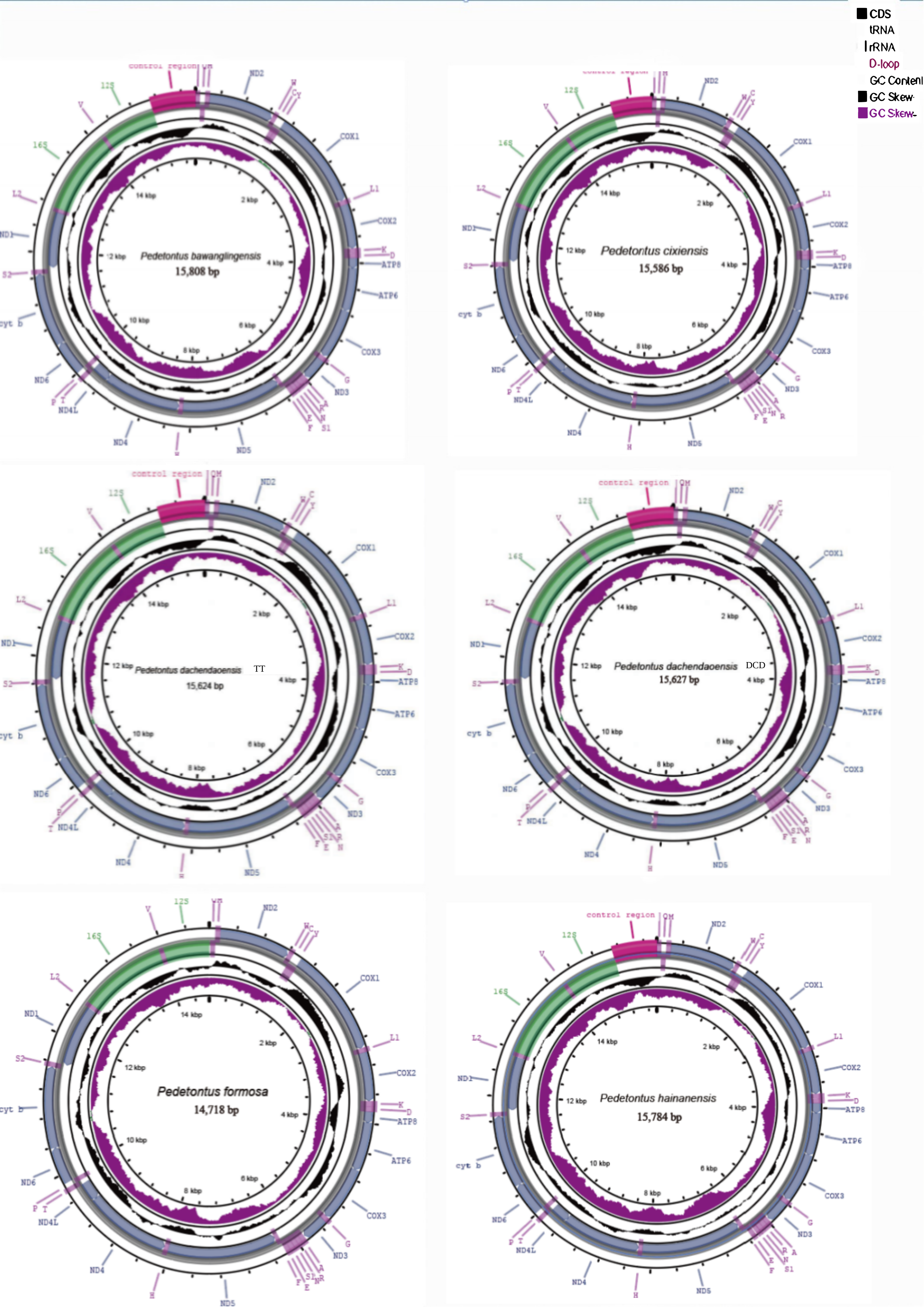
CDS
tRNA
rRNA
D-loop
GC Content
GC Skew+
GC Skew-
Pedetontus bawanglingensis
15,808 bp
Pedetontus cixiensis
15,586 bp
Pedetontus dachendaoensis TT
15,624 bp
Pedetontus dachendaoensis DCD
15,627 bp
Pedetontus formosa
14,718 bp
Pedetontus hainanensis
15,784 bp

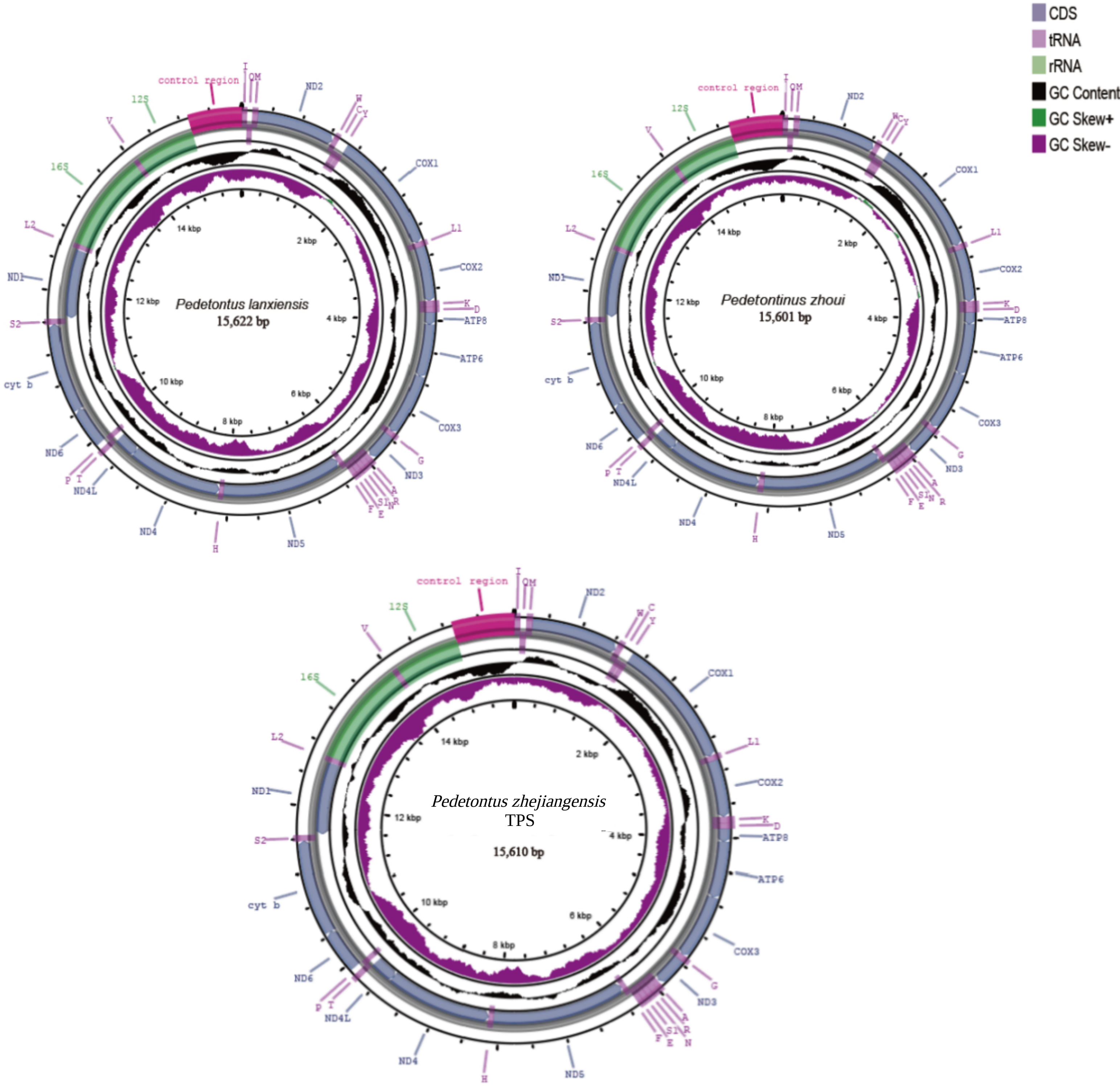
CDS
tRNA
rRNA
GC Content
GC Skew+
GC Skew-
Pedetontus lanxiensis
15,622 bp
control region
I
QM
ND2
W
C
Y
COX1
L1
COX2
K
D
ATP8
ATP6
COX3
G
ND3
A
R
N
S1
E
F
ND5
H
ND4
ND4L
T
P
ND6
cyt b
S2
ND1
L2
16S
V
12S
2 kbp
4 kbp
6 kbp
8 kbp
10 kbp
12 kbp
14 kbp
Pedetontinus zhoui
15,601 bp
Pedetontus zhejiangensis
TPS
15,610 bp
