# Supplementary material for: Comparative Mitogenomics of Pedetontus and Pedetontinus (Insecta: Archaeognatha) Unveils Phylogeny, Divergence History, and Adaptive Evolution
Source: Insects. 2025 Nov 24;16(12):1194. doi: 10.3390/insects16121194 (PMC12733737; doi:10.3390/insects16121194)
Supplement: Supplementary file 1 [file insects-16-01194-s001.zip › Figure S2 Relative Synonymous Codon Usage (RSCU) patterns in 14 mitochondrial genomes.pdf]

Figure S2.Relative Synonymous Codon Usage (RSCU) patterns in 14 mitochondrial genomes

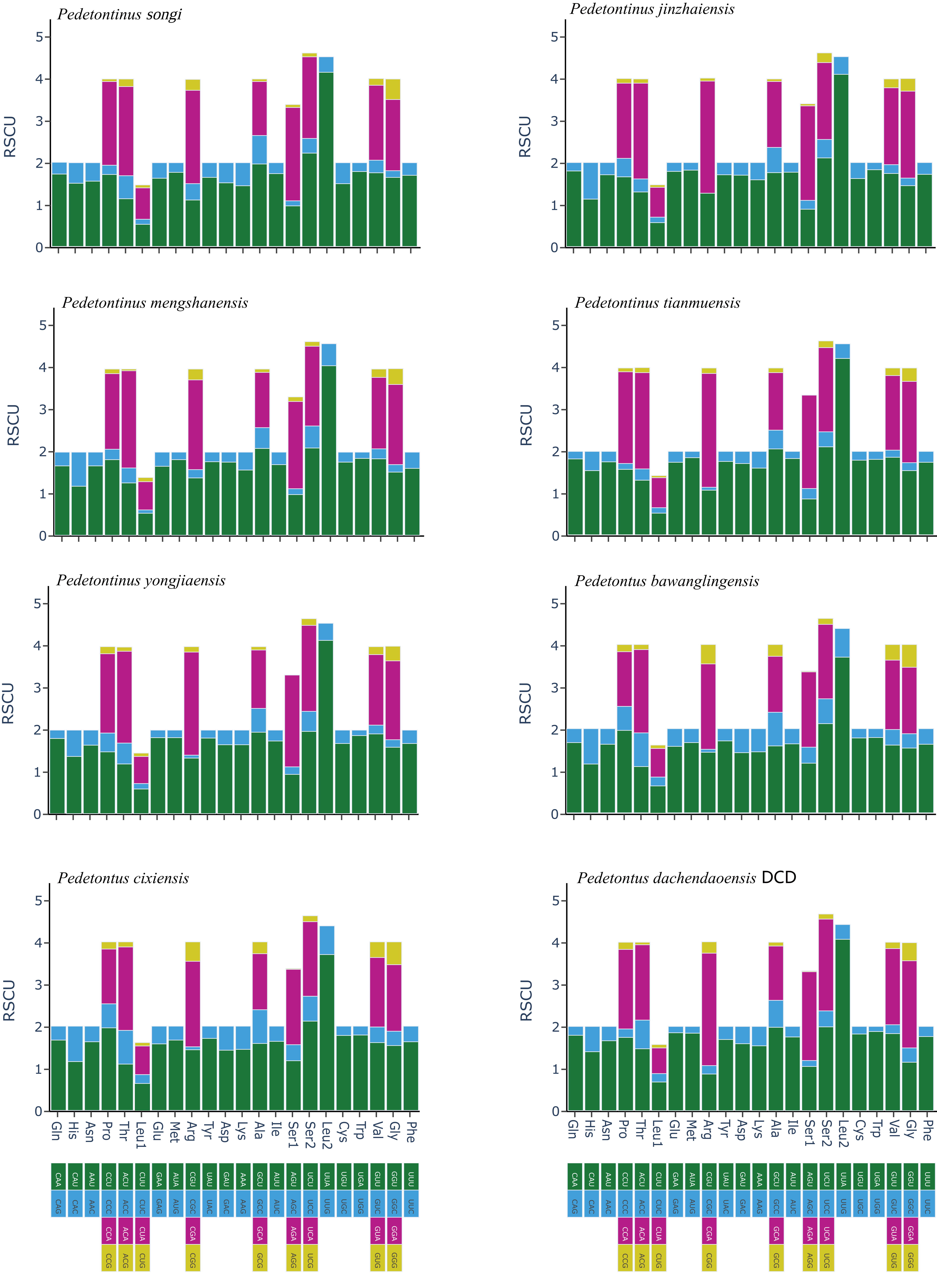

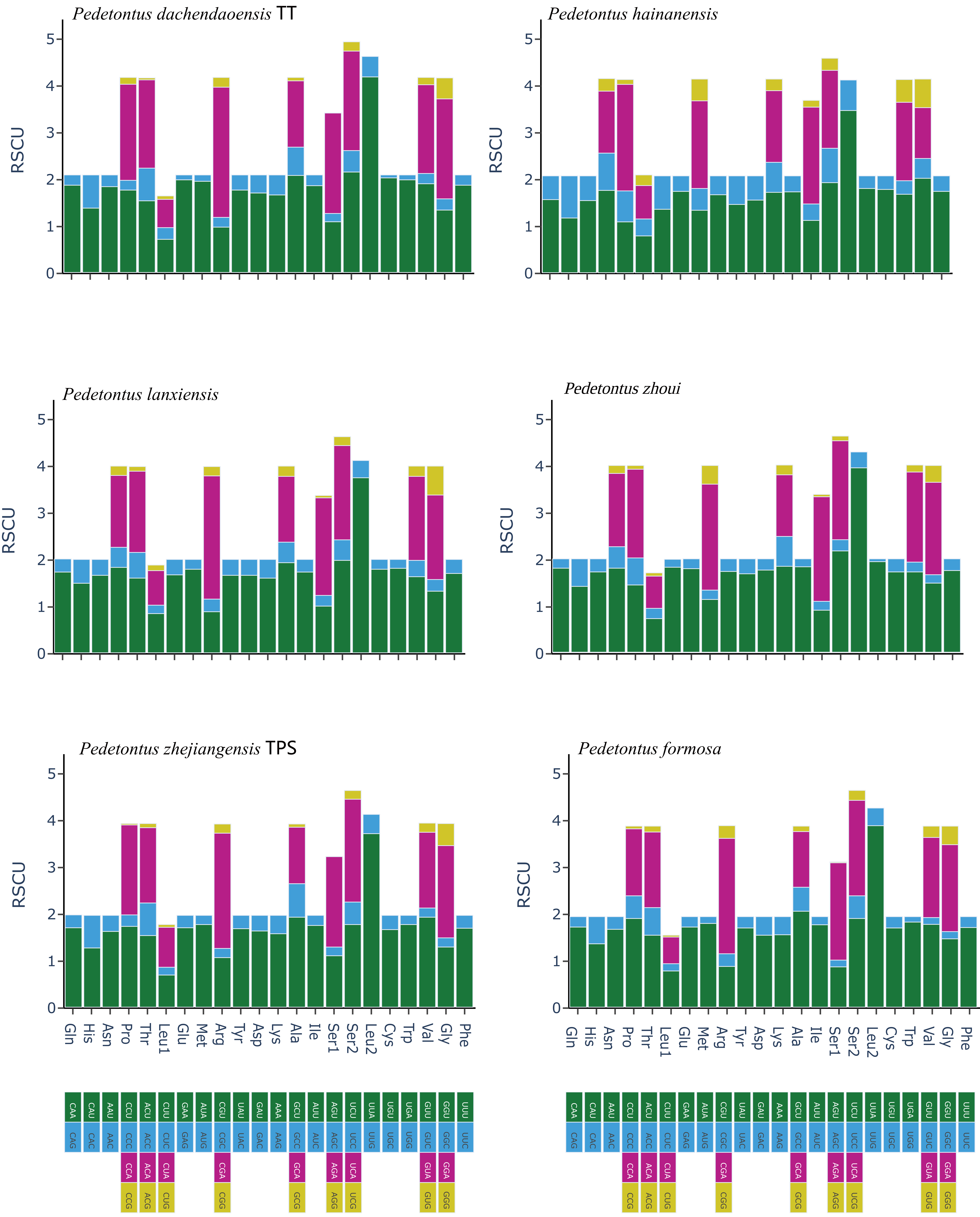

Pedetontus dachendaoensis TT
Pedetontus hainanensis
Pedetontus lanxiensis
Pedetontus zhoui
Pedetontus zhejiangensis TPS
Pedetontus formosa
RSCU
0 1 2 3 4 5
Gln His Asn Pro Thr Leu1 Glu Met Arg Tyr Asp Lys Ala Ile Ser1 Ser2 Leu2 Cys Trp Val Gly Phe
CAA CAU AAU CCU ACU CUU GAA AUA CGU UAU GAU AAA GCU AUU AGU UCU UUA UGU UGA GUU GGU UUU
CAG CAC AAC CCC ACC CUC GAG AUG CGC UAC GAC AAG GCC AUC AGC UCC UUG UGC UGG GUC GGC UUC
CCA ACA CUA CGA GCA AGA UCA GUA GGA
CCG ACG CUG CGG GCG AGG UCG GUG GGG
