# Supplementary material for: Comparative Mitogenomics of Pedetontus and Pedetontinus (Insecta: Archaeognatha) Unveils Phylogeny, Divergence History, and Adaptive Evolution
Source: Insects. 2025 Nov 24;16(12):1194. doi: 10.3390/insects16121194 (PMC12733737; doi:10.3390/insects16121194)
Supplement: Supplementary file 1 [file insects-16-01194-s001.zip › Figure S3 Inferred hairpin structures of 14 mitogenomes.pdf]

Figure S3. Inferred hairpin structures of 14 mitogenomes. The circle means the length between the *NDI* gene and *16S RNA* gene.

ND1

5'AGTAGGATAAGTCTGTTCTTTTGTAAAAAGCTTTACTGCATCAGAAAATGGTTGGGGGATA 3'

3'TCATCCTATTCAGACAAGAAAACATTTTTCGAAATGACGTAGTCTTTTACCAACCCCCTAT 5'

16S

1144bp

1. *Pedetontinus songi*

ND1

5'GATAAGTTTGTTCTTTTGTAAAAAGCTTTACTGCATCAGAAAATGGTTGAGGGATA 3'

3'CTATTCAAACAAGAAAACATTTTTCGAAATGACGTAGTCTTTTACCAACTCCCTAT 5'

16S

1146bp

2. *Pedetontinus jinzhaiensis*

ND1

5'GATAAGTTTGTTCTTTTGTAAAAAGCTTTACTGCATCAGAAAATGGTTGAGGGATA 3'

3'CTATTCAAACAAGAAAACATTTTTCGAAATGACGTAGTCTTTTACCAACTCCCTAT 5'

16S

1139bp

3. *Pedetontinus mengshanensis*

ND1

5'TTTGTAAAAAGCTTTACTGCATCAGAAAATGGTTGAGGGATA 3'

3'AAACATTTTTCGAAATGACGTAGTCTTTTACCAACTCCCTAT 5'

16S

1142bp

4. *Pedetontinus tianmuensis*

ND1

5'ATAAGTTTGTTCTTTTGTAAAAAGCTTTACTGCATCAGAAAATGGTTGAGGGATA 3'

3'TATTCAAACAAGAAAACATTTTTCGAAATGACGTAGTCTTTTACCAACTCCCTAT 5'

16S

1144bp

5. *Pedetontinus yongjiaensis*

ND1

5'TGAAATAGTTGGATAAGTTTGTTCTTTCGTAAAAAGCTTTACTGCATCAGCAAATGGTTGAGGGAT 3'

3'ACTTTATCAACCTATTCAAACAAGAAAGCATTTTTCGAAATGACGTAGTCGTTTACCAACTCCCTA 5'

16S

1154bp

6. *Pedetontus bawanglingensis*

ND1

5'TTTATTAAATAGCTTTACAGCATCAGCAAATGGTTGAGGGATC 3'

3'AAATAATTTATCGAAATGTCGTAGTCGTTTACCAACTCCCTAG 5'

16S

1143bp

7. *Pedetontus cixiensis*

ND1

5'TAGAAATAATAGGATAAGTTTGTTCTTTTGTAAAAAGCTTTACAGCATCAGCAAATGGTTGAGGGATA

3'ATCTTTATTATCCTATTCAAACAAGAAAACATTTTTCGAAATGTCGTAGTCGTTTACCAACTCCCTAT 5'

16S

1152bp

8.*Pedetontus dachendaoensis* TT

ND1

5'TAGAAATAATAGGATAACTTTGTTCTTTTGTAAAAAGCTTTACAGCATCA 3'

3'ATCTTTATTATCCTATTGAAACAAGAAAACATTTTTCGAAATGTCGTAGT 5'

16S

1190bp

9. *Pedetontus dachendaoensis DCD*

ND1

5'GTAAATAGTTAGAAGTAGTAGGATAAGTTTGTTCCTTTGTAAAAAGCTTTACAGCATCAGCAAATGGCTGAGGGAC 3'

3'CATTTATCAAT C TTCATCATCCTATTCAAACAAGGAAACATTTTTCGAAATGTCGTAGTCGTTTACCGACTCCCTG 5'

16S

1138bp

10. *Pedetontus formosa*

ND1

5'AGGATAAGTTTGTTCTTTTGTAAAAAGTTTACTGCATCAGCAAGTGGCTGAGGGATT 3'

3'TCCTATTCAAACAAGAAAACATTTTTCAAAATGACGTAGTCGTTCACCGACTCCCTAA 5'

16S

1158bp

11. *Pedetontus hainanensis*

ND1

5'TAGAAGTAGTAGGATAATTTTGTTCTTTTGTAAAAAGCTTTACAGCATCAGCAAATGGTTGAGGGAT 3'

3'ATCTTCATCATCCTATTAAAACAAGAAAACATTTTTCGAAATGTCGTAGTCGTTTACCAACTCCCTA 5'

16S

1149bp

12. *Pedetontus lanxiensis*

ND1

5'TAATTAGAAGTAGTAGGATAACTTTGTTCTTTAGTAAAAAGCTTTACAGCATCAGCAAATGGTTGAGGGAT 3'

3'ATTAATCTTCATCATCCTATTGAAACAAGAAATCATTTTTCGAAATGTCGTAGTCGTTTACCAACTCCCTA 5'

16S

1139bp

13. *Pedetontus zhoui*

ND1

5'TAGAAATAATAGGATAAGTTTGTTCTTTTGTAAAAAGCTTTACAGCATCAGCAAATGGTTGAGGGAT 3'

3'ATCTTTATTATCCTATTCAAACAAGAAAACATTTTTCGAAATGTCGTAGTCGTTTACCAACTCCCTA 5'

16S

1147bp

14. *Pedetontus zhejiangensis* TPS
