# Supplementary material for: Comparative Mitogenomics of Pedetontus and Pedetontinus (Insecta: Archaeognatha) Unveils Phylogeny, Divergence History, and Adaptive Evolution
Source: Insects. 2025 Nov 24;16(12):1194. doi: 10.3390/insects16121194 (PMC12733737; doi:10.3390/insects16121194)
Supplement: Supplementary file 1 [file insects-16-01194-s001.zip › Table S1 Species data employed in the construction of the phylogenetic tree.pdf]

Table S1. Species data employed in the construction of the phylogenetic tree.

Family	Subfamily	Genus	Species	Length(bp)	GenBank Accession Number
Meinertellidae		*Nesomachilis*	*Nesomachilis australica*	15,474 bp	NC_006895
Machilidae	Petrobiellinae	*Petrobiellus*	*Petrobiellus sp.1* JZ 2014	15,843 bp	KJ754503
			Petrobiellus sp.1 JZ 2014	14,022 bp	KJ754504
		Allopsontus	*Allopsontus sp.1* JZ 2014	15,532 bp	KJ754500
			Allopsontus sp.2 JZ 2014	15,538 bp	KJ754501
	Machilinae	*Trigoniophthalmus*	*Trigoniophthalmus alternatus*	16,197 bp	NC_010532
		Coreamachilis	*Coreamachilis coreanus*	15,578 bp	MW752137
			Coreamachilis songi	15,570 bp	MW752138
		Songmachilis	*Songmachilis xinxiangensis*	15,473 bp	NC_021384
		Machilis	*Machilis hrabei*	15,585bp	PV126557
	Petrobiinae	*Pedetontinus*	*Pedetontinus luanchuanensis*	14,106 bp	KJ754502
			Pedetontinus songi	15,631 bp	PX391408
			Pedetontinus jinzhaiensis	14,625 bp	PX391406
			Pedetontinus mengshanensis	15,654 bp	PX391407
			Pedetontinus tianmuensis	15,626 bp	PX391409
			Pedetontinus yongjiaensis	15,633 bp	PX391410
		Petrobius	*Petrobius brevistylis*	15,698 bp	NC_007688
		Pedetontus	*Pedetontus zhejiangensis*	15,602 bp	NC_051491
			Pedetontus lanxiensis	15,622 bp	PX391417
			Pedetontus formosa	14,718 bp	PX391415
			Pedetontus dachendaoensis DCD	15,627 bp	PX391414
			Pedetontus cixiensis	15,586 bp	PX391412
			Pedetontus dachendaoensis TT	15,624 bp	PX391413

		Pedetontus zhejiangensis TPS	15,610 bp	PX391418
		Pedetontus hainanensis	15,784 bp	PX391416
		Pedetontus bawanglingensis	15,808 bp	PX391411
		Pedetontus zhoui	15,601 bp	PX391419
		Pedetontus silvestrii	15,879 bp	NC_011717
		Pedetontus silvestrii DD	15,742 bp	PV126559
		Pedetontus silvestrii FC	15,872 bp	PV126560
		Pedetontus silvestrii GCL	15,705 bp	PV126561
		Pedetontus silvestrii TH	15,770 bp	PV126555
		Pedetontus silvestrii CD	15,747 bp	PV126558
		Pedetontus silvestrii XY	15,749 bp	PV126556
Collembola	outgroup	*Onychiurus orientalis*	12,984 bp	NC_006074
		Podura aquatica	13,809 bp	NC_006075
