# Supplementary material for: Comparative Mitogenomics of Pedetontus and Pedetontinus (Insecta: Archaeognatha) Unveils Phylogeny, Divergence History, and Adaptive Evolution
Source: Insects. 2025 Nov 24;16(12):1194. doi: 10.3390/insects16121194 (PMC12733737; doi:10.3390/insects16121194)
Supplement: Supplementary file 1 [file insects-16-01194-s001.zip › Table S2 The lengths of mitochondrial genes, AT content, AT skew and GC skew of 14 mitochondrial genomes.pdf]

Table S2. The lengths of mitochondrial genes, AT content, AT skew and GC skew of 14 mitochondrial genomes.

Species	Region	whole genome	PCGs		tRNA		rRNA
	Strand	Heavy	Heavy	Light	Heavy	Light	Light
Pedetontinus songi	length(bp)	15631	6900	4326	947	537	2201
	AT%	73. 6	70. 3	75	75. 6	72. 3	77. 1
	AT-skew	0. 06	−0. 035	−0. 248	0. 02	0. 026	−0. 019
	GC-skew	−0. 185	−0. 138	0. 262	−0. 004	0. 248	0. 285
Pedetontinus jinzhaiensis	length(bp)	14625	6900	4326	964	543	1868
	AT%	73. 5	70. 7	75. 9	75. 4	72. 1	77. 4
	AT-skew	0. 05	−0. 044	−0. 23	0. 015	0. 049	−0. 014
	GC-skew	−0. 182	−0. 135	0. 267	−0. 021	0. 276	0. 246
Pedetontinus mengshanensis	length(bp)	15654	6900	4320	950	545	2210
	AT%	73. 8	70. 6	75. 5	75. 6	72. 9	76. 8
	AT-skew	0. 058	−0. 045	−0. 251	0. 024	0. 018	−0. 029
	GC-skew	−0. 189	−0. 141	0. 271	0. 013	0. 257	0. 305
Pedetontinus tianmuensis	length(bp)	15626	6900	4320	951	541	2199
	AT%	74. 8	71. 8	76. 4	75. 7	73. 2	77. 2
	AT-skew	0. 051	−0. 048	−0. 243	0. 025	0. 056	−0. 009
	GC-skew	−0. 182	−0. 121	0. 289	−0. 013	0. 214	0. 261
Pedetontinus yongjiaensis	length(bp)	15633	6897	4320	953	542	2187
	AT%	73. 9	70. 6	75. 8	75. 2	72	77. 3
	AT-skew	0. 051	−0. 047	−0. 239	0. 026	0. 036	−0. 014
	GC-skew	−0. 185	−0. 134	0. 27	−0. 017	0. 263	0. 27
Pedetontus bawanglingensis	length(bp)	15808	6882	4347	971	564	2284
	AT%	71. 6	68. 5	73	73. 8	70. 6	75. 6
	AT-skew	0. 053	−0. 054	−0. 272	0. 017	0. 045	−0. 01

	GC-skew	−0.216	−0.165	0.304	−0.012	0.241	0.312
Pedetontus cixiensis	length(bp)	15586	6900	4326	955	534	2174
	AT%	74.2	71.2	76	75.6	73.8	77
	AT-skew	0.041	−0.059	−0.216	0.03	0.03	−0.037
	GC-skew	−0.225	−0.186	0.288	−0.004	0.257	0.32
Pedetontus dachendaoensis TT	length(bp)	15624	6882	4332	944	543	2170
	AT%	75	71.3	77.1	77.4	73.6	78.4
	AT-skew	0.038	−0.065	−0.206	0.015	0.005	−0.023
	GC-skew	−0.199	−0.151	0.259	−0.005	0.273	0.33
Pedetontus dachendaoensis DCD	length(bp)	15627	6882	4341	944	545	2180
	AT%	74.7	71.3	76.9	77.2	73.9	77.7
	AT-skew	0.042	−0.06	−0.209	0.01	0.012	−0.042
	GC-skew	−0.204	−0.159	0.261	0.014	0.268	0.34
Pedetontus formosa	length(bp)	14718	6879	4335	875	539	2039
	AT%	74.7	72.5	76.7	77.7	73.5	77
	AT-skew	0.023	−0.089	−0.198	0.024	0.02	−0.026
	GC-skew	−0.2	−0.15	0.263	−0.005	0.301	0.306
Pedetontus hainanensis	length(bp)	15784	6882	4317	980	571	2261
	AT%	70.9	67.8	71.9	73.3	71.5	74
	AT-skew	0.061	−0.053	−0.282	0.045	0.005	−0.019
	GC-skew	−0.24	−0.176	0.333	−0.061	0.325	0.325
Pedetontus lanxiensis	length(bp)	15622	6888	4323	942	546	2182
	AT%	74.1	70.9	75.6	76.8	73.8	76.6
	AT-skew	0.036	−0.067	−0.203	0.036	0.027	−0.044
	GC-skew	−0.213	−0.167	0.262	0.009	0.343	0.324
Pedetontus zhoui	length(bp)	15601	6885	4341	963	545	2165

	AT%	74.9	71.7	76.8	77.5	74.4	77.2
	AT-skew	0.034	−0.076	−0.201	0.024	0.03	−0.051
	GC-skew	−0.205	−0.159	0.262	−0.014	0.252	0.336
Pedetontus zhejiangensis TPS	length(bp)	15610	6876	4332	949	539	2158
	AT%	74.2	71.1	76.1	76.7	72.9	76.8
	AT-skew	0.038	−0.066	−0.208	0.033	−0.008	−0.028
	GC-skew	−0.211	−0.165	0.254	−0.005	0.329	0.343
