# Supplementary material for: Comparative Mitogenomics of Pedetontus and Pedetontinus (Insecta: Archaeognatha) Unveils Phylogeny, Divergence History, and Adaptive Evolution
Source: Insects. 2025 Nov 24;16(12):1194. doi: 10.3390/insects16121194 (PMC12733737; doi:10.3390/insects16121194)
Supplement: Supplementary file 1 [file insects-16-01194-s001.zip › Table S3 Location of features of fourteen mitogenomes.pdf]

Table S3. Location of features of fourteen mitogenomes

Pedetontinus songi

Gene	From	To	Start	Stop	Strand
trnI	1	70			H
trnQ	74	145			L
trnM	149	219			H
nad2	220	1254	ATG	TAA	H
trnW	1257	1326			H
trnC	1326	1392			L
trnY	1401	1467			L
cox1	1460	2999	ATT	T	H
trnL	3000	3064			H
cox2	3064	3751	ATG	T	H
trnK	3752	3823			H
trnD	3827	3893			H
atp8	3894	4055	ATC	TAA	H
atp6	4049	4726	ATG	TAA	H
cox3	4726	5508	ATG	TAA	H
trnG	5508	5574			H
nad3	5572	5928	ATA	TAA	H
trnA	5939	6003			H
trnR	6011	6076			H
trnN	6076	6143			H
trnS	6144	6211			H
trnE	6213	6279			H
trnF	6281	6345			L
nad5	6346	8083	ATG	T	L
trnH	8085	8148			L
nad4	8157	9506	ATG	TAA	L
nad4L	9500	9802	ATG	TAA	L
trnT	9805	9872			H
trnP	9873	9935			L
nad6	9938	10459	ATT	TAA	H
CYTB	10463	11599	ATG	TAA	H
S_copy2	11600	11662			H
nad1	11663	12598	ATA	TAA	L
L_copy2	12607	12673			L
rrnL	12674	14023			L
trnV	14024	14095			L
rrnS	14096	14946			L
control region	14947	15631			

Pedetontinus jinzhaiensis

Gene	From	To	Start	Stop	Strand
trnI	1	69			H
trnQ	74	146			L
trnM	152	223			H
nad2	224	1258	ATG	TAA	H
trnW	1262	1330			H
trnC	1331	1397			L
trnY	1406	1471			L
cox1	1464	3003	ATT	T	H
trnL	3004	3069			H
cox2	3070	3757	GTG	T	H
trnK	3758	3829			H
trnD	3834	3902			H
atp8	3903	4064	ATC	TAA	H
atp6	4058	4735	ATG	TAA	H
cox3	4735	5517	ATG	TAA	H
trnG	5517	5583			H
nad3	5581	5937	ATA	TAA	H
trnA	5938	6012			H
trnR	6018	6083			H
trnN	6083	6149			H
trnS	6150	6217			H
trnE	6219	6287			H
trnF	6289	6354			L
nad5	6355	8092	ATG	T	L
trnH	8094	8162			L
nad4	8166	9515	ATG	TAA	L
nad4L	9509	9811	ATG	TAA	L
trnT	9814	9881			H
trnP	9882	9944			L
nad6	9947	10468	ATT	TAA	H
CYTB	10472	11608	ATG	TAA	H
S_copy2	11607	11673			H
nad1	11674	12609	ATA	TAG	L
L_copy2	12619	12685			L
rrnL	12686	14032			L
trnV	14033	14104			L
rrnS	14105	14625			L

Pedetontinus mengshanensis

Gene	From	To	Start	Stop	Strand
trnI	1	69			H
trnQ	73	146			L
trnM	151	221			H
nad2	222	1256	ATG	TAA	H
trnW	1260	1329			H
trnC	1329	1396			L
trnY	1407	1473			L
cox1	1466	3005	ATT	T	H
trnL	3006	3070			H
cox2	3071	3758	GTG	T	H
trnK	3759	3830			H
trnD	3837	3905			H
atp8	3906	4067	ATC	TAA	H
atp6	4061	4738	ATG	TAA	H
cox3	4738	5520	ATG	TAA	H
trnG	5520	5585			H
nad3	5583	5939	ATA	TAA	H
trnA	5950	6011			H
trnR	6015	6084			H
trnN	6084	6151			H
trnS	6152	6219			H
trnE	6221	6286			H
trnF	6288	6353			L
nad5	6354	8091	ATG	T	L
trnH	8093	8157			L
nad4	8161	9510	ATG	TAA	L
nad4L	9504	9806	ATG	TAA	L
trnT	9809	9875			H
trnP	9876	9940			L
nad6	9943	10464	ATT	TAA	H
CYTB	10468	11604	ATG	TAA	H
S_copy2	11603	11669			H
nad1	11668	12597	ATA	TAA	L
L_copy2	12605	12671			L
rrnL	12672	14012			L
trnV	14013	14085			L
rrnS	14086	14954			L
control region	14955	15654			

Pedetontinus tianmuensis

Gene	From	To	Start	Stop	Strand
trnI	1	69			H
trnQ	74	146			L
trnM	148	219			H
nad2	220	1254	ATG	TAA	H
trnW	1258	1326			H
trnC	1326	1392			L
trnY	1399	1465			L
cox1	1458	2997	ATT	T	H
trnL	2998	3064			H
cox2	3065	3752	GTG	T	H
trnK	3753	3824			H
trnD	3829	3896			H
atp8	3897	4058	ATT	TAA	H
atp6	4052	4729	ATG	TAA	H
cox3	4729	5511	ATG	TAA	H
trnG	5511	5578			H
nad3	5576	5932	ATA	TAA	H
trnA	5944	6008			H
trnR	6015	6079			H
trnN	6080	6145			H
trnS	6146	6213			H
trnE	6215	6282			H
trnF	6284	6349			L
nad5	6350	8087	ATG	T	L
trnH	8089	8153			L
nad4	8157	9506	ATG	TAA	L
nad4L	9500	9802	ATG	TAA	L
trnT	9805	9871			H
trnP	9872	9935			L
nad6	9938	10459	ATT	TAA	H
CYTB	10463	11599	ATG	TAA	H
S_copy2	11598	11664			H
nad1	11667	12596	ATA	TAG	L
L_copy2	12606	12672			L
rrnL	12673	14011			L
trnV	14012	14083			L
rrnS	14084	14943			L
control region	14944	15626			

Pedetontinus yongjiaensis

Gene	From	To	Start	Stop	Strand
trnI	1	69			H
trnQ	73	145			L
trnM	150	221			H
nad2	222	1256	ATG	TAA	H
trnW	1260	1328			H
trnC	1328	1396			L
trnY	1405	1470			L
cox1	1463	3002	ATT	T	H
trnL	3003	3069			H
cox2	3070	3757	GTG	T	H
trnK	3758	3829			H
trnD	3832	3900			H
atp8	3901	4062	ATC	TAA	H
atp6	4056	4733	ATG	TAA	H
cox3	4733	5515	ATG	TAA	H
trnG	5515	5581			H
nad3	5582	5935	ATA	TAA	H
trnA	5949	6011			H
trnR	6017	6082			H
trnN	6083	6149			H
trnS	6150	6217			H
trnE	6219	6287			H
trnF	6289	6354			L
nad5	6355	8092	ATG	T	L
trnH	8094	8158			L
nad4	8162	9511	ATG	TAA	L
nad4L	9505	9807	ATG	TAA	L
trnT	9810	9877			H
trnP	9878	9941			L
nad6	9944	10465	ATT	TAA	H
CYTB	10469	11605	ATG	TAA	H
S_copy2	11604	11670			H
nad1	11670	12599	ATA	TAA	L
L_copy2	12609	12675			L
rrnL	12676	14018			L
trnV	14019	14090			L
rrnS	14091	14934			L
control region	14935	15633			

Pedetontus bawanglingensis

Gene	From	To	Start	Stop	Strand
trnI	1	68			H
trnQ	80	155			L
trnM	155	225			H
nad2	226	1254	GTG	TAA	H
trnW	1260	1326			H
trnC	1326	1398			L
trnY	1399	1469			L
cox1	1462	3001	ATT	T	H
trnL	3002	3071			H
cox2	3075	3762	ATG	T	H
trnK	3763	3834			H
trnD	3841	3912			H
atp8	3913	4074	ATT	TAA	H
atp6	4068	4745	ATG	TAA	H
cox3	4745	5527	ATG	TAA	H
trnG	5535	5603			H
nad3	5604	5957	ATA	TAA	H
trnA	5968	6033			H
trnR	6039	6111			H
trnN	6113	6180			H
trnS	6181	6248			H
trnE	6250	6319			H
trnF	6322	6388			L
nad5	6385	8125	ATG	T	L
trnH	8127	8196			L
nad4	8196	9545	ATG	TAA	L
nad4L	9539	9841	ATG	TAA	L
trnT	9844	9913			H
trnP	9914	9983			L
nad6	9987	10499	ATC	TAA	H
CYTB	10504	11640	ATG	TAA	H
S_copy2	11639	11705			H
nad1	11719	12672	ATA	TAA	L
L_copy2	12670	12734			L
rrnL	12735	14116			L
trnV	14117	14188			L
rrnS	14189	15090			L
control region	15091	15808			

Pedetontus cixiensis

Gene	From	To	Start	Stop	Strand
trnI	1	70			H
trnQ	72	142			L
trnM	142	213			H
nad2	214	1254	ATA	TAA	H
trnW	1254	1324			H
trnC	1327	1390			L
trnY	1391	1461			L
cox1	1454	2993	ATT	T	H
trnL	2994	3056			H
cox2	3057	3744	ATC	T	H
trnK	3745	3818			H
trnD	3820	3886			H
atp8	3887	4048	ATT	TAA	H
atp6	4042	4719	ATG	TAA	H
cox3	4719	5499	ATG	T	H
trnG	5500	5569			H
nad3	5570	5923	ATA	TAA	H
trnA	5936	5997			H
trnR	6005	6069			H
trnN	6070	6135			H
trnS	6136	6205			H
trnE	6208	6276			H
trnF	6284	6347			L
nad5	6345	8085	ATG	T	L
trnH	8087	8150			L
nad4	8148	9498	ATG	T	L
nad4L	9492	9794	ATG	TAA	L
trnT	9797	9863			H
trnP	9865	9928			L
nad6	9932	10453	ATT	TAA	H
CYTB	10453	11589	ATG	TAA	H
S_copy2	11588	11656			H
nad1	11676	12608	ATG	TAA	L
L_copy2	12612	12676			L
rrnL	12677	14018			L
trnV	14019	14089			L
rrnS	14090	14921			L
control region	14922	15586			

Pedetontus dachendaoensis DCD

Gene	From	To	Start	Stop	Strand
trnI	1	69			H
trnQ	71	145			L
trnM	145	214			H
nad2	215	1249	GTG	TAA	H
trnW	1256	1323			H
trnC	1324	1387			L
trnY	1402	1467			L
cox1	1460	2999	ATT	T	H
trnL	3000	3064			H
cox2	3065	3752	ATT	T	H
trnK	3753	3825			H
trnD	3827	3894			H
atp8	3895	4056	ATT	TAA	H
atp6	4050	4727	ATG	TAA	H
cox3	4727	5507	ATG	T	H
trnG	5508	5573			H
nad3	5574	5927	ATA	TAA	H
trnA	5945	6008			H
trnR	6013	6079			H
trnN	6082	6147			H
trnS	6148	6216			H
trnE	6218	6282			H
trnF	6284	6348			L
nad5	6349	8086	ATG	T	L
trnH	8088	8153			L
nad4	8154	9501	ATG	T	L
nad4L	9495	9797	ATG	TAA	L
trnT	9800	9866			H
trnP	9869	9937			L
nad6	9944	10453	ATA	TAA	H
CYTB	10457	11593	ATG	TAA	H
S_copy2	11592	11658			H
nad1	11664	12617	ATT	TAA	L
L_copy2	12617	12681			L
rrnL	12682	14035			L
trnV	14036	14110			L
rrnS	14111	14936			L
Control region	14937	15627			

Pedetontus dachendaoensis TT

Gene	From	To	Start	Stop	Strand
trnI	1	69			H
trnQ	71	144			L
trnM	144	213			H
nad2	214	1248	GTG	TAA	H
trnW	1254	1321			H
trnC	1322	1384			L
trnY	1400	1465			L
cox1	1458	2997	ATT	T	H
trnL	2998	3062			H
cox2	3063	3750	ATT	T	H
trnK	3751	3823			H
trnD	3826	3893			H
atp8	3894	4055	ATT	TAA	H
atp6	4049	4726	ATG	TAA	H
cox3	4726	5506	ATG	T	H
trnG	5507	5572			H
nad3	5573	5926	ATA	TAA	H
trnA	5940	6003			H
trnR	6008	6074			H
trnN	6077	6142			H
trnS	6143	6211			H
trnE	6213	6277			H
trnF	6279	6343			L
nad5	6344	8081	ATG	T	L
trnH	8083	8148			L
nad4	8148	9496	ATG	TA	L
nad4L	9490	9792	ATG	TAA	L
trnT	9795	9861			H
trnP	9864	9932			L
nad6	9939	10448	ATA	TAA	H
CYTB	10452	11588	ATG	TAA	H
S_copy2	11587	11653			H
nad1	11659	12603	ATA	TAA	L
L_copy2	12613	12677			L
rrnL	12678	14028			L
trnV	14029	14103			L
rrnS	14104	14922			L
control region	14923	15624			

Pedetontus hainanensis

Gene	From	To	Start	Stop	Strand
trnI	1	69			H
trnQ	73	148			L
trnM	152	223			H
nad2	224	1252	GTG	TAG	H
trnW	1258	1325			H
trnC	1325	1394			L
trnY	1395	1470			L
cox1	1463	3002	ATT	T	H
trnL	3003	3068			H
cox2	3072	3759	ATG	T	H
trnK	3760	3831			H
trnD	3838	3909			H
atp8	3910	4071	ATT	TAA	H
atp6	4065	4742	ATG	TAA	H
cox3	4742	5524	ATG	TAG	H
trnG	5529	5597			H
nad3	5598	5951	ATA	TAG	H
trnA	5961	6027			H
trnR	6029	6103			H
trnN	6105	6175			H
trnS	6172	6239			H
trnE	6258	6331			H
trnF	6334	6401			L
nad5	6398	8138	ATG	T	L
trnH	8140	8209			L
nad4	8209	9558	ATG	TAA	L
nad4L	9552	9854	ATG	TAA	L
trnT	9857	9927			H
trnP	9929	9997			L
nad6	10002	10514	ATA	TAA	H
CYTB	10519	11655	ATG	TAA	H
S_copy2	11654	11719			H
nad1	11750	12673	ATG	TAA	L
L_copy2	12683	12750			L
rrnL	12751	14123			L
trnV	14124	14197			L
rrnS	14198	15085			L
control region	15086	15784			

Pedetontus lanxiensis

Gene	From	To	Start	Stop	Strand
trnI	1	68			H
trnQ	71	141			L
trnM	141	211			H
nad2	212	1249	GTG	TAA	H
trnW	1253	1320			H
trnC	1320	1388			L
trnY	1398	1465			L
cox1	1458	2997	ATT	T	H
trnL	2998	3062			H
cox2	3063	3750	ATC	T	H
trnK	3751	3823			H
trnD	3826	3893			H
atp8	3894	4055	ATT	TAA	H
atp6	4049	4726	ATG	TAA	H
cox3	4726	5508	ATG	TAA	H
trnG	5508	5573			H
nad3	5574	5927	ATA	TAA	H
trnA	5939	6000			H
trnR	6004	6068			H
trnN	6071	6137			H
trnS	6138	6206			H
trnE	6207	6271			H
trnF	6272	6337			L
nad5	6338	8075	ATG	T	L
trnH	8076	8140			L
nad4	8141	9488	ATG	T	L
nad4L	9482	9784	ATG	TAA	L
trnT	9787	9853			H
trnP	9856	9921			L
nad6	9928	10437	ATA	TAA	H
CYTB	10441	11577	ATG	TAA	H
S_copy2	11576	11643			H
nad1	11649	12584	ATT	TAA	L
L_copy2	12603	12669			L
rrnL	12670	14023			L
trnV	14024	14097			L
rrnS	14098	14925			L
control region	14926	15622			

Pedetontus zhoui

Gene	From	To	Start	Stop	Strand
trnI	1	68			H
trnQ	70	141			L
trnM	141	210			H
nad2	211	1245	ATG	TAA	H
trnW	1250	1317			H
trnC	1317	1387			L
trnY	1394	1461			L
cox1	1454	2993	ATT	T	H
trnL	2994	3058			H
cox2	3059	3746	ATT	T	H
trnK	3747	3819			H
trnD	3824	3892			H
atp8	3893	4054	ATT	TAA	H
atp6	4048	4725	ATG	TAA	H
cox3	4725	5507	ATG	TAA	H
trnG	5507	5572			H
nad3	5573	5926	ATA	TAA	H
trnA	5924	6005			H
trnR	6008	6074			H
trnN	6076	6142			H
trnS	6143	6212			H
trnE	6213	6277			H
trnF	6278	6342			L
nad5	6343	8080	ATG	T	L
trnH	8082	8147			L
nad4	8147	9496	ATG	TAA	L
nad4L	9490	9792	ATG	TAA	L
trnT	9795	9859			H
trnP	9862	9925			L
nad6	9932	10441	ATA	TAA	H
CYTB	10445	11581	ATG	TAA	H
S_copy2	11580	11647			H
nad1	11653	12603	ATT	TAA	L
L_copy2	12607	12671			L
rrnL	12672	14009			L
trnV	14010	14083			L
rrnS	14084	14910			L
control region	14911	15601			

Pedetontus zhejiangensis TPS

Gene	From	To	Start	Stop	Strand
trnI	1	69			H
trnQ	71	145			L
trnM	145	214			H
nad2	215	1244	GTG	T	H
trnW	1246	1315			H
trnC	1315	1379			L
trnY	1391	1457			L
cox1	1450	2989	ATT	T	H
trnL	2990	3054			H
cox2	3055	3742	ATT	T	H
trnK	3743	3815			H
trnD	3818	3885			H
atp8	3886	4047	ATT	TAA	H
atp6	4041	4718	ATG	TAA	H
cox3	4718	5498	ATG	T	H
trnG	5499	5564			H
nad3	5565	5918	ATA	TAA	H
trnA	5933	5996			H
trnR	6002	6068			H
trnN	6070	6136			H
trnS	6137	6205			H
trnE	6207	6271			H
trnF	6272	6336			L
nad5	6337	8074	ATG	T	L
trnH	8076	8140			L
nad4	8140	9488	ATG	TA	L
nad4L	9482	9784	ATG	TAA	L
trnT	9787	9853			H
trnP	9856	9918			L
nad6	9925	10434	ATA	TAG	H
CYTB	10438	11574	ATG	TAA	H
S_copy2	11573	11641			H
nad1	11649	12593	ATA	TAA	L
L_copy2	12603	12667			L
rrnL	12668	14007			L
trnV	14008	14081			L
rrnS	14082	14899			L
control region	14900	15610			

Pedetontus formosa

Gene	From	To	Start	Stop	Strand
trnQ	20	91			L
trnM	91	160			H
nad2	161	1190	GTG	T	H
trnW	1192	1259			H
trnC	1259	1325			L
trnY	1333	1398			L
cox1	1391	2930	ATT	T	H
trnL	2931	2995			H
cox2	2996	3683	ATT	T	H
trnK	3684	3756			H
trnD	3760	3830			H
atp8	3831	3992	ATT	TAA	H
atp6	3986	4663	ATG	TAA	H
cox3	4663	5445	ATG	TAA	H
trnG	5445	5509			H
nad3	5510	5863	ATA	TAA	H
trnA	5877	5940			H
trnR	5943	6005			H
trnN	6007	6073			H
trnS	6074	6143			H
trnE	6145	6209			H
trnF	6210	6273			L
nad5	6274	8011	ATG	T	L
trnH	8013	8079			L
nad4	8079	9428	ATG	TAG	L
nad4L	9422	9724	ATG	TAA	L
trnT	9727	9793			H
trnP	9796	9859			L
nad6	9866	10375	ATA	TAA	H
CYTB	10380	11516	ATG	TAA	H
S_copy2	11515	11581			H
nad1	11587	12531	ATA	TAA	L
L_copy2	12541	12605			L
rrnL	12606	13942			L
trnV	13943	14016			L
rrnS	14017	14718			L
