# Supplementary material for: Comparative Mitogenomics of Pedetontus and Pedetontinus (Insecta: Archaeognatha) Unveils Phylogeny, Divergence History, and Adaptive Evolution
Source: Insects. 2025 Nov 24;16(12):1194. doi: 10.3390/insects16121194 (PMC12733737; doi:10.3390/insects16121194)
Supplement: Supplementary file 1 [file insects-16-01194-s001.zip › Table S4 Comparative analysis of codon usage frequency profiles among 14 mitochondrial genomes.pdf]

Table S4. Comparative analysis of codon usage frequency profiles among 14 mitochondrial genomes.

Pedetontinus songi

Codon	Count	RSCU	Codon	Count	RSCU	Codon	Count	RSCU	Codon	Count	RSCU
UUU(F)	270	1.69	UCU(S)	97	2.24	UAU(Y)	127	1.67	UGU(C)	30	1.58
UUC(F)	49	0.31	UCC(S)	16	0.37	UAC(Y)	25	0.33	UGC(C)	8	0.42
UUA(L)	390	4.13	UCA(S)	85	1.96	UAA(*)	10	2	UGA(W)	96	1.83
UUG(L)	35	0.37	UCG(S)	5	0.12	UAG(*)	0	0	UGG(W)	9	0.17
CUU(L)	50	0.53	CCU(P)	58	1.65	CAU(H)	58	1.53	CGU(R)	17	1.11
CUC(L)	12	0.13	CCC(P)	8	0.23	CAC(H)	18	0.47	CGC(R)	6	0.39
CUA(L)	73	0.77	CCA(P)	73	2.07	CAA(Q)	69	1.73	CGA(R)	34	2.23
CUG(L)	6	0.06	CCG(P)	2	0.06	CAG(Q)	11	0.28	CGG(R)	4	0.26
AUU(I)	292	1.74	ACU(T)	55	1.11	AAU(N)	128	1.53	AGU(S)	41	0.95
AUC(I)	43	0.26	ACC(T)	28	0.56	AAC(N)	39	0.47	AGC(S)	6	0.14
AUA(M)	230	1.78	ACA(T)	107	2.15	AAA(K)	62	1.46	AGA(S)	96	2.21
AUG(M)	29	0.22	ACG(T)	9	0.18	AAG(K)	23	0.54	AGG(S)	1	0.02
GUU(V)	102	1.75	GCU(A)	94	1.96	GAU(D)	54	1.54	GGU(G)	95	1.65
GUC(V)	17	0.29	GCC(A)	33	0.69	GAC(D)	16	0.46	GGC(G)	10	0.17
GUA(V)	105	1.8	GCA(A)	62	1.29	GAA(E)	62	1.63	GGA(G)	98	1.7
GUG(V)	9	0.15	GCG(A)	3	0.06	GAG(E)	14	0.37	GGG(G)	28	0.48

Pedetontinus jinzhaiensis

Codon	Count	RSCU	Codon	Count	RSCU	Codon	Count	RSCU	Codon	Count	RSCU
UUU(F)	281	1.72	UCU(S)	95	2.15	UAU(Y)	130	1.7	UGU(C)	32	1.73
UUC(F)	46	0.28	UCC(S)	19	0.43	UAC(Y)	23	0.3	UGC(C)	5	0.27
UUA(L)	390	4.09	UCA(S)	82	1.86	UAA(*)	9	1.8	UGA(W)	98	1.87
UUG(L)	40	0.42	UCG(S)	10	0.23	UAG(*)	1	0.2	UGG(W)	7	0.13
CUU(L)	54	0.57	CCU(P)	57	1.62	CAU(H)	42	1.14	CGU(R)	19	1.27
CUC(L)	12	0.13	CCC(P)	15	0.43	CAC(H)	32	0.86	CGC(R)	0	0
CUA(L)	70	0.73	CCA(P)	65	1.84	CAA(Q)	72	1.82	CGA(R)	40	2.67
CUG(L)	6	0.06	CCG(P)	4	0.11	CAG(Q)	7	0.18	CGG(R)	1	0.07
AUU(I)	300	1.78	ACU(T)	61	1.28	AAU(N)	142	1.71	AGU(S)	39	0.88
AUC(I)	37	0.22	ACC(T)	16	0.34	AAC(N)	24	0.29	AGC(S)	10	0.23
AUA(M)	234	1.83	ACA(T)	109	2.28	AAA(K)	68	1.6	AGA(S)	98	2.22
AUG(M)	22	0.17	ACG(T)	5	0.1	AAG(K)	17	0.4	AGG(S)	0	0
GUU(V)	100	1.74	GCU(A)	82	1.74	GAU(D)	62	1.7	GGU(G)	82	1.44
GUC(V)	13	0.23	GCC(A)	30	0.63	GAC(D)	11	0.3	GGC(G)	11	0.19
GUA(V)	105	1.83	GCA(A)	74	1.57	GAA(E)	69	1.79	GGA(G)	117	2.06
GUG(V)	12	0.21	GCG(A)	3	0.06	GAG(E)	8	0.21	GGG(G)	17	0.3

Pedetontinus mengshanensis

Codon	Count	RSCU	Codon	Count	RSCU	Codon	Count	RSCU	Codon	Count	RSCU
UUU(F)	267	1.62	UCU(S)	92	2.08	UAU(Y)	141	1.77	UGU(C)	30	1.76
UUC(F)	62	0.38	UCC(S)	25	0.56	UAC(Y)	18	0.23	UGC(C)	4	0.24
UUA(L)	388	4.09	UCA(S)	87	1.97	UAA(*)	10	2	UGA(W)	97	1.85
UUG(L)	49	0.52	UCG(S)	5	0.11	UAG(*)	0	0	UGG(W)	8	0.15
CUU(L)	52	0.55	CCU(P)	66	1.82	CAU(H)	46	1.21	CGU(R)	21	1.4
CUC(L)	8	0.08	CCC(P)	9	0.25	CAC(H)	30	0.79	CGC(R)	3	0.2
CUA(L)	63	0.66	CCA(P)	66	1.82	CAA(Q)	66	1.67	CGA(R)	33	2.2
CUG(L)	9	0.09	CCG(P)	4	0.11	CAG(Q)	13	0.33	CGG(R)	3	0.2
AUU(I)	285	1.71	ACU(T)	64	1.32	AAU(N)	132	1.66	AGU(S)	43	0.97
AUC(I)	48	0.29	ACC(T)	17	0.35	AAC(N)	27	0.34	AGC(S)	6	0.14
AUA(M)	234	1.82	ACA(T)	111	2.29	AAA(K)	69	1.57	AGA(S)	92	2.08
AUG(M)	23	0.18	ACG(T)	2	0.04	AAG(K)	19	0.43	AGG(S)	4	0.09
GUU(V)	101	1.84	GCU(A)	100	2.07	GAU(D)	65	1.76	GGU(G)	87	1.54
GUC(V)	13	0.24	GCC(A)	24	0.5	GAC(D)	9	0.24	GGC(G)	10	0.18
GUA(V)	94	1.72	GCA(A)	65	1.35	GAA(E)	64	1.66	GGA(G)	108	1.91
GUG(V)	11	0.2	GCG(A)	4	0.08	GAG(E)	13	0.34	GGG(G)	21	0.37

Pedetontinus tianmuensis

Codon	Count	RSCU	Codon	Count	RSCU	Codon	Count	RSCU	Codon	Count	RSCU
UUU(F)	291	1.74	UCU(S)	93	2.11	UAU(Y)	134	1.76	UGU(C)	34	1.79
UUC(F)	44	0.26	UCC(S)	16	0.36	UAC(Y)	18	0.24	UGC(C)	4	0.21
UUA(L)	404	4.23	UCA(S)	89	2.02	UAA(*)	9	1.8	UGA(W)	95	1.81
UUG(L)	33	0.35	UCG(S)	7	0.16	UAG(*)	1	0.2	UGG(W)	10	0.19
CUU(L)	50	0.52	CCU(P)	54	1.57	CAU(H)	57	1.54	CGU(R)	16	1.07
CUC(L)	12	0.13	CCC(P)	5	0.14	CAC(H)	17	0.46	CGC(R)	1	0.07
CUA(L)	69	0.72	CCA(P)	76	2.2	CAA(Q)	71	1.82	CGA(R)	41	2.73
CUG(L)	5	0.05	CCG(P)	3	0.09	CAG(Q)	7	0.18	CGG(R)	2	0.13
AUU(I)	318	1.83	ACU(T)	64	1.31	AAU(N)	140	1.75	AGU(S)	38	0.86
AUC(I)	30	0.17	ACC(T)	13	0.27	AAC(N)	20	0.25	AGC(S)	11	0.25
AUA(M)	232	1.84	ACA(T)	113	2.31	AAA(K)	69	1.6	AGA(S)	99	2.24
AUG(M)	20	0.16	ACG(T)	6	0.12	AAG(K)	17	0.4	AGG(S)	0	0
GUU(V)	101	1.86	GCU(A)	96	2.06	GAU(D)	65	1.71	GGU(G)	87	1.54
GUC(V)	9	0.17	GCC(A)	21	0.45	GAC(D)	11	0.29	GGC(G)	11	0.19
GUA(V)	97	1.79	GCA(A)	64	1.38	GAA(E)	67	1.74	GGA(G)	110	1.95
GUG(V)	10	0.18	GCG(A)	5	0.11	GAG(E)	10	0.26	GGG(G)	18	0.32

Pedetontinus yongjiaensis

Codon	Count	RSCU	Codon	Count	RSCU	Codon	Count	RSCU	Codon	Count	RSCU
UUU(F)	275	1.68	UCU(S)	86	1.97	UAU(Y)	138	1.8	UGU(C)	32	1.78
UUC(F)	52	0.32	UCC(S)	21	0.48	UAC(Y)	15	0.2	UGC(C)	4	0.22
UUA(L)	398	4.15	UCA(S)	90	2.06	UAA(*)	10	2	UGA(W)	100	1.9
UUG(L)	39	0.41	UCG(S)	7	0.16	UAG(*)	0	0	UGG(W)	5	0.1
CUU(L)	56	0.58	CCU(P)	52	1.48	CAU(H)	51	1.38	CGU(R)	20	1.33
CUC(L)	12	0.13	CCC(P)	16	0.45	CAC(H)	23	0.62	CGC(R)	1	0.07
CUA(L)	62	0.65	CCA(P)	67	1.9	CAA(Q)	71	1.8	CGA(R)	37	2.47
CUG(L)	8	0.08	CCG(P)	6	0.17	CAG(Q)	8	0.2	CGG(R)	2	0.13
AUU(I)	285	1.74	ACU(T)	57	1.19	AAU(N)	135	1.64	AGU(S)	42	0.96
AUC(I)	43	0.26	ACC(T)	24	0.5	AAC(N)	30	0.36	AGC(S)	7	0.16
AUA(M)	229	1.82	ACA(T)	105	2.2	AAA(K)	71	1.67	AGA(S)	96	2.2
AUG(M)	23	0.18	ACG(T)	5	0.1	AAG(K)	14	0.33	AGG(S)	0	0
GUU(V)	113	1.93	GCU(A)	96	1.98	GAU(D)	61	1.65	GGU(G)	91	1.59
GUC(V)	12	0.21	GCC(A)	27	0.56	GAC(D)	13	0.35	GGC(G)	10	0.17
GUA(V)	98	1.68	GCA(A)	67	1.38	GAA(E)	71	1.82	GGA(G)	108	1.89
GUG(V)	11	0.19	GCG(A)	4	0.08	GAG(E)	7	0.18	GGG(G)	20	0.35

Pedetontus bawanglingensis

Codon	Count	RSCU	Codon	Count	RSCU	Codon	Count	RSCU	Codon	Count	RSCU
UUU(F)	265	1.62	UCU(S)	89	2.13	UAU(Y)	137	1.73	UGU(C)	34	1.79
UUC(F)	62	0.38	UCC(S)	24	0.57	UAC(Y)	21	0.27	UGC(C)	4	0.21
UUA(L)	360	3.72	UCA(S)	73	1.74	UAA(*)	10	2	UGA(W)	92	1.79
UUG(L)	66	0.68	UCG(S)	6	0.14	UAG(*)	0	0	UGG(W)	11	0.21
CUU(L)	63	0.65	CCU(P)	71	2	CAU(H)	47	1.16	CGU(R)	22	1.44
CUC(L)	20	0.21	CCC(P)	20	0.56	CAC(H)	34	0.84	CGC(R)	1	0.07
CUA(L)	64	0.66	CCA(P)	45	1.27	CAA(Q)	61	1.67	CGA(R)	31	2.03
CUG(L)	8	0.08	CCG(P)	6	0.17	CAG(Q)	12	0.33	CGG(R)	7	0.46
AUU(I)	260	1.64	ACU(T)	55	1.11	AAU(N)	127	1.63	AGU(S)	50	1.19
AUC(I)	58	0.36	ACC(T)	39	0.79	AAC(N)	29	0.37	AGC(S)	17	0.41
AUA(M)	209	1.66	ACA(T)	98	1.98	AAA(K)	66	1.45	AGA(S)	76	1.81
AUG(M)	43	0.34	ACG(T)	6	0.12	AAG(K)	25	0.55	AGG(S)	0	0
GUU(V)	91	1.61	GCU(A)	80	1.61	GAU(D)	58	1.45	GGU(G)	91	1.54
GUC(V)	21	0.37	GCC(A)	39	0.78	GAC(D)	22	0.55	GGC(G)	20	0.34
GUA(V)	93	1.65	GCA(A)	66	1.33	GAA(E)	62	1.59	GGA(G)	92	1.56
GUG(V)	21	0.37	GCG(A)	14	0.28	GAG(E)	16	0.41	GGG(G)	33	0.56

Pedetontus cixiensis

Codon	Count	RSCU	Codon	Count	RSCU	Codon	Count	RSCU	Codon	Count	RSCU
UUU(F)	287	1.71	UCU(S)	86	2.03	UAU(Y)	125	1.64	UGU(C)	34	1.74
UUC(F)	49	0.29	UCC(S)	14	0.33	UAC(Y)	27	0.36	UGC(C)	5	0.26
UUA(L)	353	3.77	UCA(S)	87	2.05	UAA(*)	8	2	UGA(W)	93	1.77
UUG(L)	35	0.37	UCG(S)	10	0.24	UAG(*)	0	0	UGG(W)	12	0.23
CUU(L)	71	0.76	CCU(P)	62	1.82	CAU(H)	55	1.38	CGU(R)	22	1.47
CUC(L)	17	0.18	CCC(P)	15	0.44	CAC(H)	25	0.62	CGC(R)	6	0.4
CUA(L)	79	0.84	CCA(P)	57	1.68	CAA(Q)	70	1.87	CGA(R)	32	2.13
CUG(L)	7	0.07	CCG(P)	2	0.06	CAG(Q)	5	0.13	CGG(R)	0	0
AUU(I)	334	1.81	ACU(T)	74	1.52	AAU(N)	137	1.57	AGU(S)	49	1.16
AUC(I)	35	0.19	ACC(T)	31	0.64	AAC(N)	37	0.43	AGC(S)	11	0.26
AUA(M)	241	1.79	ACA(T)	87	1.78	AAA(K)	87	1.71	AGA(S)	82	1.94
AUG(M)	29	0.21	ACG(T)	3	0.06	AAG(K)	15	0.29	AGG(S)	0	0
GUU(V)	95	1.79	GCU(A)	78	2	GAU(D)	52	1.55	GGU(G)	62	1.12
GUC(V)	11	0.21	GCC(A)	23	0.59	GAC(D)	15	0.45	GGC(G)	21	0.38
GUA(V)	92	1.74	GCA(A)	51	1.31	GAA(E)	72	1.73	GGA(G)	114	2.05
GUG(V)	14	0.26	GCG(A)	4	0.1	GAG(E)	11	0.27	GGG(G)	25	0.45

Pedetontus dachendaoensis DCD

Codon	Count	RSCU	Codon	Count	RSCU	Codon	Count	RSCU	Codon	Count	RSCU
UUU(F)	294	1.76	UCU(S)	84	2.01	UAU(Y)	129	1.69	UGU(C)	30	1.82
UUC(F)	40	0.24	UCC(S)	16	0.38	UAC(Y)	24	0.31	UGC(C)	3	0.18
UUA(L)	399	4.06	UCA(S)	91	2.17	UAA(*)	8	2	UGA(W)	94	1.88
UUG(L)	36	0.37	UCG(S)	5	0.12	UAG(*)	0	0	UGG(W)	6	0.12
CUU(L)	67	0.68	CCU(P)	61	1.74	CAU(H)	51	1.4	CGU(R)	13	0.87
CUC(L)	20	0.2	CCC(P)	7	0.2	CAC(H)	22	0.6	CGC(R)	3	0.2
CUA(L)	60	0.61	CCA(P)	66	1.89	CAA(Q)	67	1.79	CGA(R)	40	2.67
CUG(L)	8	0.08	CCG(P)	6	0.17	CAG(Q)	8	0.21	CGG(R)	4	0.27
AUU(I)	309	1.75	ACU(T)	69	1.47	AAU(N)	150	1.66	AGU(S)	45	1.07
AUC(I)	45	0.25	ACC(T)	32	0.68	AAC(N)	31	0.34	AGC(S)	6	0.14
AUA(M)	251	1.85	ACA(T)	84	1.79	AAA(K)	74	1.56	AGA(S)	87	2.08
AUG(M)	21	0.15	ACG(T)	3	0.06	AAG(K)	21	0.44	AGG(S)	1	0.02
GUU(V)	93	1.81	GCU(A)	84	1.98	GAU(D)	57	1.58	GGU(G)	64	1.15
GUC(V)	11	0.21	GCC(A)	27	0.64	GAC(D)	15	0.42	GGC(G)	19	0.34
GUA(V)	94	1.83	GCA(A)	55	1.29	GAA(E)	74	1.85	GGA(G)	115	2.07
GUG(V)	8	0.16	GCG(A)	4	0.09	GAG(E)	6	0.15	GGG(G)	24	0.43

Pedetontus dachendaoensis TT

Codon	Count	RSCU	Codon	Count	RSCU	Codon	Count	RSCU	Codon	Count	RSCU
UUU(F)	296	1.79	UCU(S)	85	2.06	UAU(Y)	127	1.69	UGU(C)	33	1.94
UUC(F)	35	0.21	UCC(S)	18	0.44	UAC(Y)	23	0.31	UGC(C)	1	0.06
UUA(L)	392	4.01	UCA(S)	84	2.04	UAA(*)	8	2	UGA(W)	95	1.9
UUG(L)	41	0.42	UCG(S)	8	0.19	UAG(*)	0	0	UGG(W)	5	0.1
CUU(L)	67	0.68	CCU(P)	60	1.69	CAU(H)	49	1.32	CGU(R)	14	0.93
CUC(L)	23	0.24	CCC(P)	7	0.2	CAC(H)	25	0.68	CGC(R)	3	0.2
CUA(L)	57	0.58	CCA(P)	70	1.97	CAA(Q)	67	1.79	CGA(R)	40	2.67
CUG(L)	7	0.07	CCG(P)	5	0.14	CAG(Q)	8	0.21	CGG(R)	3	0.2
AUU(I)	315	1.78	ACU(T)	70	1.47	AAU(N)	162	1.76	AGU(S)	43	1.04
AUC(I)	39	0.22	ACC(T)	32	0.67	AAC(N)	22	0.24	AGC(S)	7	0.17
AUA(M)	250	1.86	ACA(T)	86	1.81	AAA(K)	77	1.59	AGA(S)	85	2.06
AUG(M)	19	0.14	ACG(T)	2	0.04	AAG(K)	20	0.41	AGG(S)	0	0
GUU(V)	94	1.82	GCU(A)	86	1.99	GAU(D)	58	1.63	GGU(G)	71	1.28
GUC(V)	11	0.21	GCC(A)	25	0.58	GAC(D)	13	0.37	GGC(G)	13	0.23
GUA(V)	94	1.82	GCA(A)	59	1.36	GAA(E)	76	1.9	GGA(G)	114	2.05
GUG(V)	8	0.15	GCG(A)	3	0.07	GAG(E)	4	0.1	GGG(G)	24	0.43

Pedetontus hainanensis

Codon	Count	RSCU	Codon	Count	RSCU	Codon	Count	RSCU	Codon	Count	RSCU
UUU(F)	266	1.65	UCU(S)	80	1.9	UAU(Y)	126	1.63	UGU(C)	32	1.78
UUC(F)	56	0.35	UCC(S)	31	0.74	UAC(Y)	29	0.37	UGC(C)	4	0.22
UUA(L)	333	3.45	UCA(S)	67	1.59	UAA(*)	7	1.4	UGA(W)	90	1.76
UUG(L)	58	0.6	UCG(S)	11	0.26	UAG(*)	3	0.6	UGG(W)	12	0.24
CUU(L)	72	0.75	CCU(P)	61	1.74	CAU(H)	43	1.12	CGU(R)	18	1.2
CUC(L)	32	0.33	CCC(P)	26	0.74	CAC(H)	34	0.88	CGC(R)	7	0.47
CUA(L)	65	0.67	CCA(P)	44	1.26	CAA(Q)	56	1.51	CGA(R)	29	1.93
CUG(L)	19	0.2	CCG(P)	9	0.26	CAG(Q)	18	0.49	CGG(R)	6	0.4
AUU(I)	255	1.67	ACU(T)	58	1.14	AAU(N)	125	1.54	AGU(S)	47	1.12
AUC(I)	51	0.33	ACC(T)	32	0.63	AAC(N)	37	0.46	AGC(S)	12	0.28
AUA(M)	215	1.69	ACA(T)	110	2.16	AAA(K)	61	1.51	AGA(S)	86	2.04
AUG(M)	39	0.31	ACG(T)	4	0.08	AAG(K)	20	0.49	AGG(S)	3	0.07
GUU(V)	98	1.61	GCU(A)	78	1.63	GAU(D)	57	1.39	GGU(G)	117	1.94
GUC(V)	18	0.3	GCC(A)	31	0.65	GAC(D)	25	0.61	GGC(G)	23	0.38
GUA(V)	100	1.64	GCA(A)	71	1.49	GAA(E)	49	1.29	GGA(G)	66	1.1
GUG(V)	28	0.46	GCG(A)	11	0.23	GAG(E)	27	0.71	GGG(G)	35	0.58

Pedetontus lanxiensis

Codon	Count	RSCU	Codon	Count	RSCU	Codon	Count	RSCU	Codon	Count	RSCU
UUU(F)	280	1.71	UCU(S)	86	2.05	UAU(Y)	121	1.67	UGU(C)	34	1.89
UUC(F)	47	0.29	UCC(S)	18	0.43	UAC(Y)	24	0.33	UGC(C)	2	0.11
UUA(L)	372	3.8	UCA(S)	85	2.03	UAA(*)	9	2	UGA(W)	92	1.84
UUG(L)	35	0.36	UCG(S)	7	0.17	UAG(*)	0	0	UGG(W)	8	0.16
CUU(L)	80	0.82	CCU(P)	64	1.83	CAU(H)	53	1.47	CGU(R)	13	0.88
CUC(L)	17	0.17	CCC(P)	15	0.43	CAC(H)	19	0.53	CGC(R)	4	0.27
CUA(L)	73	0.74	CCA(P)	54	1.54	CAA(Q)	71	1.73	CGA(R)	39	2.64
CUG(L)	11	0.11	CCG(P)	7	0.2	CAG(Q)	11	0.27	CGG(R)	3	0.2
AUU(I)	315	1.72	ACU(T)	78	1.61	AAU(N)	146	1.66	AGU(S)	40	0.96
AUC(I)	51	0.28	ACC(T)	27	0.56	AAC(N)	30	0.34	AGC(S)	9	0.21
AUA(M)	244	1.83	ACA(T)	84	1.73	AAA(K)	77	1.62	AGA(S)	89	2.13
AUG(M)	23	0.17	ACG(T)	5	0.1	AAG(K)	18	0.38	AGG(S)	1	0.02
GUU(V)	81	1.6	GCU(A)	80	1.9	GAU(D)	60	1.67	GGU(G)	73	1.31
GUC(V)	17	0.34	GCC(A)	19	0.45	GAC(D)	12	0.33	GGC(G)	15	0.27
GUA(V)	92	1.82	GCA(A)	60	1.43	GAA(E)	68	1.68	GGA(G)	102	1.83
GUG(V)	12	0.24	GCG(A)	9	0.21	GAG(E)	13	0.32	GGG(G)	33	0.59

Pedetontus zhoui

Codon	Count	RSCU	Codon	Count	RSCU	Codon	Count	RSCU	Codon	Count	RSCU
UUU(F)	297	1.76	UCU(S)	91	2.19	UAU(Y)	130	1.72	UGU(C)	34	1.94
UUC(F)	41	0.24	UCC(S)	10	0.24	UAC(Y)	21	0.28	UGC(C)	1	0.06
UUA(L)	389	3.96	UCA(S)	89	2.14	UAA(*)	10	2	UGA(W)	86	1.72
UUG(L)	33	0.34	UCG(S)	4	0.1	UAG(*)	0	0	UGG(W)	14	0.28
CUU(L)	71	0.72	CCU(P)	62	1.8	CAU(H)	52	1.41	CGU(R)	17	1.13
CUC(L)	22	0.22	CCC(P)	16	0.46	CAC(H)	22	0.59	CGC(R)	3	0.2
CUA(L)	68	0.69	CCA(P)	54	1.57	CAA(Q)	71	1.8	CGA(R)	34	2.27
CUG(L)	7	0.07	CCG(P)	6	0.17	CAG(Q)	8	0.2	CGG(R)	6	0.4
AUU(I)	334	1.83	ACU(T)	69	1.43	AAU(N)	160	1.72	AGU(S)	38	0.91
AUC(I)	31	0.17	ACC(T)	28	0.58	AAC(N)	26	0.28	AGC(S)	8	0.19
AUA(M)	239	1.79	ACA(T)	92	1.91	AAA(K)	78	1.75	AGA(S)	92	2.21
AUG(M)	28	0.21	ACG(T)	4	0.08	AAG(K)	11	0.25	AGG(S)	1	0.02
GUU(V)	83	1.71	GCU(A)	78	1.84	GAU(D)	57	1.68	GGU(G)	81	1.46
GUC(V)	10	0.21	GCC(A)	27	0.64	GAC(D)	11	0.32	GGC(G)	10	0.18
GUA(V)	94	1.94	GCA(A)	56	1.32	GAA(E)	73	1.82	GGA(G)	110	1.98
GUG(V)	7	0.14	GCG(A)	9	0.21	GAG(E)	7	0.17	GGG(G)	21	0.38

Pedetontus zhejiangensis TPS

Codon	Count	RSCU	Codon	Count	RSCU	Codon	Count	RSCU	Codon	Count	RSCU
UUU(F)	289	1.72	UCU(S)	74	1.82	UAU(Y)	124	1.7	UGU(C)	27	1.69
UUC(F)	48	0.28	UCC(S)	20	0.49	UAC(Y)	22	0.3	UGC(C)	5	0.31
UUA(L)	373	3.78	UCA(S)	90	2.21	UAA(*)	6	1.71	UGA(W)	91	1.8
UUG(L)	41	0.42	UCG(S)	8	0.2	UAG(*)	1	0.29	UGG(W)	10	0.2
CUU(L)	69	0.7	CCU(P)	63	1.76	CAU(H)	47	1.29	CGU(R)	16	1.08
CUC(L)	18	0.18	CCC(P)	9	0.25	CAC(H)	26	0.71	CGC(R)	3	0.2
CUA(L)	85	0.86	CCA(P)	70	1.96	CAA(Q)	69	1.73	CGA(R)	37	2.51
CUG(L)	6	0.06	CCG(P)	1	0.03	CAG(Q)	11	0.28	CGG(R)	3	0.2
AUU(I)	312	1.78	ACU(T)	72	1.57	AAU(N)	151	1.65	AGU(S)	46	1.13
AUC(I)	39	0.22	ACC(T)	32	0.7	AAC(N)	32	0.35	AGC(S)	7	0.17
AUA(M)	248	1.79	ACA(T)	76	1.65	AAA(K)	77	1.6	AGA(S)	81	1.99
AUG(M)	29	0.21	ACG(T)	4	0.09	AAG(K)	19	0.4	AGG(S)	0	0
GUU(V)	100	1.96	GCU(A)	83	1.95	GAU(D)	58	1.66	GGU(G)	73	1.31
GUC(V)	10	0.2	GCC(A)	31	0.73	GAC(D)	12	0.34	GGC(G)	11	0.2
GUA(V)	84	1.65	GCA(A)	53	1.25	GAA(E)	71	1.73	GGA(G)	112	2.01
GUG(V)	10	0.2	GCG(A)	3	0.07	GAG(E)	11	0.27	GGG(G)	27	0.48

Pedetontus formosa

Codon	Count	RSCU	Codon	Count	RSCU	Codon	Count	RSCU	Codon	Count	RSCU
UUU(F)	299	1.77	UCU(S)	82	2	UAU(Y)	140	1.78	UGU(C)	29	1.76
UUC(F)	38	0.23	UCC(S)	20	0.49	UAC(Y)	17	0.22	UGC(C)	4	0.24
UUA(L)	395	4.03	UCA(S)	86	2.1	UAA(*)	8	1.78	UGA(W)	96	1.94
UUG(L)	37	0.38	UCG(S)	8	0.2	UAG(*)	1	0.22	UGG(W)	3	0.06
CUU(L)	80	0.82	CCU(P)	67	1.97	CAU(H)	50	1.41	CGU(R)	13	0.88
CUC(L)	14	0.14	CCC(P)	17	0.5	CAC(H)	21	0.59	CGC(R)	4	0.27
CUA(L)	58	0.59	CCA(P)	50	1.47	CAA(Q)	66	1.76	CGA(R)	38	2.58
CUG(L)	4	0.04	CCG(P)	2	0.06	CAG(Q)	9	0.24	CGG(R)	4	0.27
AUU(I)	337	1.84	ACU(T)	74	1.58	AAU(N)	167	1.73	AGU(S)	36	0.88
AUC(I)	29	0.16	ACC(T)	28	0.6	AAC(N)	26	0.27	AGC(S)	6	0.15
AUA(M)	256	1.86	ACA(T)	78	1.67	AAA(K)	77	1.66	AGA(S)	89	2.17
AUG(M)	19	0.14	ACG(T)	7	0.15	AAG(K)	16	0.34	AGG(S)	1	0.02
GUU(V)	87	1.8	GCU(A)	88	2.11	GAU(D)	54	1.59	GGU(G)	84	1.5
GUC(V)	8	0.17	GCC(A)	22	0.53	GAC(D)	14	0.41	GGC(G)	9	0.16
GUA(V)	85	1.76	GCA(A)	51	1.22	GAA(E)	71	1.77	GGA(G)	108	1.93
GUG(V)	13	0.27	GCG(A)	6	0.14	GAG(E)	9	0.23	GGG(G)	23	0.41
