# Supplementary material for: Comparative Mitogenomics of Pedetontus and Pedetontinus (Insecta: Archaeognatha) Unveils Phylogeny, Divergence History, and Adaptive Evolution
Source: Insects. 2025 Nov 24;16(12):1194. doi: 10.3390/insects16121194 (PMC12733737; doi:10.3390/insects16121194)
Supplement: Supplementary file 1 [file insects-16-01194-s001.zip › Table S5 Genetic distance analysis of the COI gene among species of Pedetontinus and Pedetontus included in this study.pdf]

Table S5. Genetic distance analysis of the *COI* gene among species of *Pedetontinus* and *Pedetontus* included in this study. Species corresponding to numbers 1–23 are listed below: 1: *Pn. songi*; 2: *Pn. jinzhaiensis*; 3: *Pn. luanchuanensis* KJ754502; 4: *Pn. mengshanensis*; 5: *Pn. tianmuensis*; 6: *Pn. yongjiaensis*; 7: *Pd. bawanglingensis*; 8: *Pd. cixiensis*; 9: *Pd. dachendaoensis* TT; 10: *Pd. Dachendaoensis* DCD; 11: *Pd. formosa*; 12: *Pd. hainanensis*; 13: *Pd. lanxiensis*; 14: *Pd. silvestrii* CD PV126558; 15: *Pd. silvestrii* DD PV126559; 16: *Pd. silvestrii* FC PV126560; 17: *Pd. silvestrii* GCL PV126561; 18: *Pd. silvestrii* TH PV126555; 19: *Pd. silvestrii* XY PV126556; 20: *Pd. silvestrii* NC_011717; 21: *Pd. zhoui*; 22: *Pd. zhejiangensis* TPS; 23: *Pd. zhejiangensis* NC_051491.

	1	*2*	*3*	*4*	*5*	*6*	*7*	*8*	*9*	*10*	*11*	*12*	*13*	*14*	*15*	*16*	*17*	*18*	*19*	*20*	*21*	*22*
1																						
2	0.109																					
3	0.142	0.117																				
4	0.125	0.101	0.129																			
5	0.115	0.079	0.121	0.107																		
6	0.116	0.074	0.135	0.109	0.103																	
7	0.196	0.189	0.201	0.191	0.193	0.201																
8	0.214	0.193	0.210	0.197	0.213	0.204	0.215															
9	0.192	0.175	0.208	0.183	0.180	0.186	0.213	0.184														
10	0.194	0.171	0.202	0.181	0.176	0.184	0.211	0.179	0.036													
11	0.195	0.177	0.194	0.186	0.187	0.192	0.209	0.184	0.120	0.122												
12	0.175	0.179	0.194	0.172	0.169	0.183	0.161	0.206	0.207	0.202	0.215											
13	0.205	0.192	0.204	0.188	0.205	0.208	0.214	0.194	0.116	0.115	0.130	0.211										
14	0.213	0.185	0.210	0.192	0.205	0.199	0.224	0.181	0.180	0.186	0.174	0.230	0.181									
15	0.200	0.188	0.213	0.193	0.191	0.192	0.206	0.181	0.166	0.163	0.162	0.209	0.183	0.148								
16	0.203	0.189	0.205	0.191	0.194	0.191	0.208	0.172	0.163	0.161	0.156	0.211	0.176	0.146	0.015							
17	0.206	0.196	0.230	0.198	0.208	0.215	0.229	0.187	0.176	0.173	0.185	0.226	0.198	0.160	0.142	0.139						
18	0.213	0.209	0.226	0.201	0.209	0.205	0.219	0.183	0.181	0.176	0.173	0.213	0.191	0.166	0.090	0.085	0.150					
19	0.206	0.198	0.213	0.188	0.199	0.200	0.218	0.185	0.173	0.177	0.163	0.212	0.183	0.151	0.062	0.064	0.158	0.102				

20	0.213	0.183	0.214	0.198	0.201	0.199	0.227	0.185	0.189	0.186	0.173	0.221	0.177	0.097	0.151	0.149	0.168	0.169	0.158			
21	0.205	0.194	0.199	0.189	0.201	0.204	0.214	0.193	0.117	0.123	0.109	0.205	0.128	0.179	0.163	0.161	0.195	0.182	0.170	0.171		
22	0.194	0.186	0.205	0.186	0.185	0.186	0.203	0.185	0.100	0.102	0.115	0.205	0.120	0.174	0.165	0.161	0.184	0.182	0.178	0.172	0.126	
23	0.199	0.192	0.212	0.195	0.201	0.198	0.210	0.179	0.099	0.105	0.116	0.211	0.127	0.182	0.165	0.162	0.192	0.176	0.171	0.187	0.131	0.051
