# Supplementary material for: Comparative Mitogenomics of Pedetontus and Pedetontinus (Insecta: Archaeognatha) Unveils Phylogeny, Divergence History, and Adaptive Evolution
Source: Insects. 2025 Nov 24;16(12):1194. doi: 10.3390/insects16121194 (PMC12733737; doi:10.3390/insects16121194)
Supplement: Supplementary file 1 [file insects-16-01194-s001.zip › Table S6 Pairwise genetic distance values among Pedetontus and Pedetontinus groups.pdf]

Table S6. Pairwise genetic distance values among *Pedetontus* and *Pedetontinus* groups.

	1	2	3	4
Group 1<br>(*Pn. songi,*<br>*Pn. jinzhaiensis,*<br>*Pn. mengshanensis,*<br>*Pn. tianmuensis,*<br>*Pn. yongjiaensis,*<br>*Pn. luanchuanensis*（KJ754502）)				
Group 2<br>(*Pd. hainanensis,*<br>*Pd. bawanglingensis*)	0.187			
Group 3<br>(*Pd. cixiensis*)	0.205	0.211		
Group 4<br>(*Pd. zhejiangensis* TPS*,*<br>*Pd. zhoui,*<br>*Pd. lanxiensis,*<br>*Pd. formosa,*<br>*Pd. Dachendaoensis* DCD*,*<br>*Pd. dachendaoensis* TT*,*<br>*Pd. zhejiangensis*（NC_051491）)	0.193	0.209	0.186	
Group 5<br>(*Pd. silvestrii*<br>XY/TH/GCL/FC/DD/CD/ref（NC_011717）)	0.202	0.218	0.182	0.175

	1	2
Group 1<br>All *Pedetontinus*		
Group 2<br>All *Pedetontus*<br>(excluding *Pd. hainanensis* and *Pd. bawanglingensis*)	0.198	
